# A critical period of susceptibility to sound in the sensory cells of cephalopod hatchlings.

**DOI:** 10.1242/bio.033860

**Published:** 2018-10-05

**Authors:** Marta Solé, Marc Lenoir, José-Manuel Fortuño, Mike van der Schaar, Michel André

**Affiliations:** 1 Laboratory of Applied Bioacoustics (LAB), Technical University of Catalonia, Vilanova i la Geltrú. 08800. Barcelona Tech (UPC), Spain; 2 Department of Physiopathology and Therapy of Sensory and Motor Deficits INSERM U.1051, Institute of Neurosciences of Montpellier, 34000 Montpellier, France; 3 Electron Microscopy Laboratory, Institute of Marine Sciences, Spanish National Research Council, E-08003 Barcelona, Spain

**Keywords:** Cephalopod hatchlings, Statocyst, Sensory hair cell, Lateral line system, Acoustic impact, Anthropogenic noise, Electron microscopy

## Abstract

The cephalopod statocyst and lateral line systems are sensory organs involved in orientation and balance. Lateral lines allow cephalopods to detect particle motion and are used for locating prey or predators in low light conditions. Here, we show the first analysis of damaged sensory epithelia in three species of cephalopod hatchlings (*Sepia officinalis*, *Loligo vulgaris* and *Illex coindetii*) after sound exposure. Our results indicate lesions in the statocyst sensory epithelia, similar to what was found in adult specimens. The novelty is that the severity of the lesions advanced more rapidly in hatchlings than in adult animals; i.e. the degree of lesions seen in hatchlings immediately after noise exposure would develop within 48 h in adults. This feature suggests a critical period of increased sensitivity to acoustic trauma in those species as has been described in developing mammalian cochlea and avian basilar papilla. The hair cells in the lateral lines of *S. officinalis* followed the same pattern of damage occurrence, while those of *L. vulgaris* and *I. coindetii* displayed a decreasing severity of damage after 24 h. These differences could be due to dissimilarities in size and life stages between the three species.

## Abstract (precis)

**Summary:** We provide evidence of acoustic trauma in cephalopod hatchling sensory cells after sound exposure, whose damage increases faster than in adults, suggesting a critical period of sensitivity to anthropogenic noise in early stages.

## INTRODUCTION

There is a considerable lack of information concerning the cephalopod's reception of sounds (Packard et al., 1990; Bleckmann et al., 1991; Bullock and Budelmann, 1991; Budelmann et al., 1995; Hu et al., 2009; Kaifu et al., 2008; Mooney et al., 2010). Cephalopods are sensitive to vibration stimuli and are able to perceive these stimuli through the statocyst receptor and the lateral line analogue systems (Budelmann et al., 1997; Budelmann and Bleckmann, 1988). Several authors have addressed studies on invertebrate sensitivity to noise and possible negative effects after sound exposure (Budelmann and Bleckmann, 1988; Lagardère, 1982; Regnault and Lagardere, 1983; Lovell et al., 2005, 2006; Fewtrell and McCauley, 2012; Aguilar de Soto, 2016; Carroll et al., 2017). A detailed literature review on the inner structure of the statocyst and the effect of sound on its sensory epithelia can be found in recent publications (André et al., 2011; Solé et al., 2013a,b, 2016, 2017).

Many cephalopods have lines of ciliated cells on their head and arms which are considered invertebrate analogue to the mechanoreceptive lateral lines of fish and aquatic amphibians. This is an example of convergent evolution between a sophisticated cephalopod and a vertebrate sensory system (Budelmann and Bleckmann, 1988). The ciliated cells of this lateral line system are sensitive to local water movements and are able to perceive hydrodynamic pressure. The lines of epidermal hair cells running over the head and arms of the cephalopod are shown to be able to detect local water movements generated by a vibrating sphere (Budelmann and Bleckmann, 1988; Komak et al., 2005). Stimulation of the lines with artificial water displacements of defined frequency and amplitude evoke receptor potentials with features very similar to the lateral microphonic potential of fish (Bleckmann et al., 1991; Budelmann et al., 1997; Budelmann and Bleckmann, 1988).

The epidermal lines are present in the late embryonic stages and hatchlings of cephalopods (Naef, 1928; Sundermann, 1982, 1983; Lenz et al., 1995; Lenz, 1997; Villanueva and Norman, 2008). This sensory system consists of ciliated primary sensory hair cells that carry kinocilia with an internal 9×2+2 tubules content (Sundermann, 1983; Hanlon and Budelmann, 1987) and non-ciliated accessory cells, running in an anterior-posterior direction, located on the arms, head, anterior part of dorsal mantle and funnel. Cuttlefish and squid show eight epidermal lines on their head (four on each side), two dorsally- and two laterally-positioned lines (one above and one below the eye), which continue on the arms (except the line below the eye, Fig. S1), an additional paired, short, fifth line on the ventral side of the head (Sundermann, 1983) and a broad band of ciliated cells on the ventral funnel surface.

In *Octopus vulgaris* hatchlings, the unique single funnel line is located along its midline. The other epidermal lines (dorsal, dorsoventral, ventrolateral and ventral) are paired occupying both sides of the head and right and left arms (Fig. S1). As opposed to cuttlefish and squid, the epidermal lines found in octopus paralarvae have not been reported in adult octopi.

Each epidermal line consists of ciliated cells that carry approximately 100 kinocilia per cell, each 10–20 µm in length. Each hair cell is surrounded by several smaller supporting cells that carry only microvilli (Sundermann, 1983). Each hair cell is polarized in one direction, according to the orientation of the cilia's basal feet and the 9×2+2 tubules content (Budelmann et al., 1997). The cells are primary sensory hair cells and their axons run underneath them (Sundermann, 1983).

The effects of sound on the functionality and the physiology of the cephalopods statocyst, as a consequence of an exposure to artificial noise, are reviewed in previous publications (André et al., 2011; Solé et al., 2013a,b, 2017). However, despite some references in the literature on the effects of sound exposure on the lateral lines of fish, no mention could be found of the analogous lateral line of cephalopods. The lateral line system of fish consists of a set of receptors, located at the body surface, which detect water motion close to the fish. The lateral line has been shown to be important to predatory fish in locating prey and to prey fish in mediating escape behaviour (Coombs and Montgomery, 1999). Although Hastings et al. (1996) suggested there were no effects on sensory cells of the lateral line of *Astronotus ocellatus* after sound exposure, Denton and Gray (1993) showed that mechanical stimulation of the lateral line of clupeids may cause damage by decoupling the cupulae from the neuromasts (sensory structures with hair cells) of the lateral line. A loss of the attachment between the cupula and the neuromast would result in a dysfunction of the lateral line system.

Data on the effects of sound on fish larva are very scarce as well. Kostyuchenko (1973) reported damage on neuromasts of the lateral line system in cod (*Gadus morhua*) and Atlantic herring (*Clupea harengus*) larva under seismic air-gun sound exposure. Booman et al. (1996) investigated the effects of seismic air guns on eggs and larva in different marine species (Atlantic cod, herring, saithe). These authors also described damage to neuromasts of the lateral line system and other organ systems. Advanced studies adressing the effects of sound on the lateral line in fishes and cephalopods, specially on larvae are necessary.

Here, we conducted controlled exposure experiments on the Mediterranean *S. officinalis*, *L. vulgaris* and *I. coindetii* hatchlings to look at potential effects of sound overexposure on the ciliated primary sensory hair cells in both the epidermal lines and in the statocyst of the three species.

## RESULTS

### Structural and ultrastructural analysis of the epidermal lines’ sensory epithelium

**Fig. 1.**
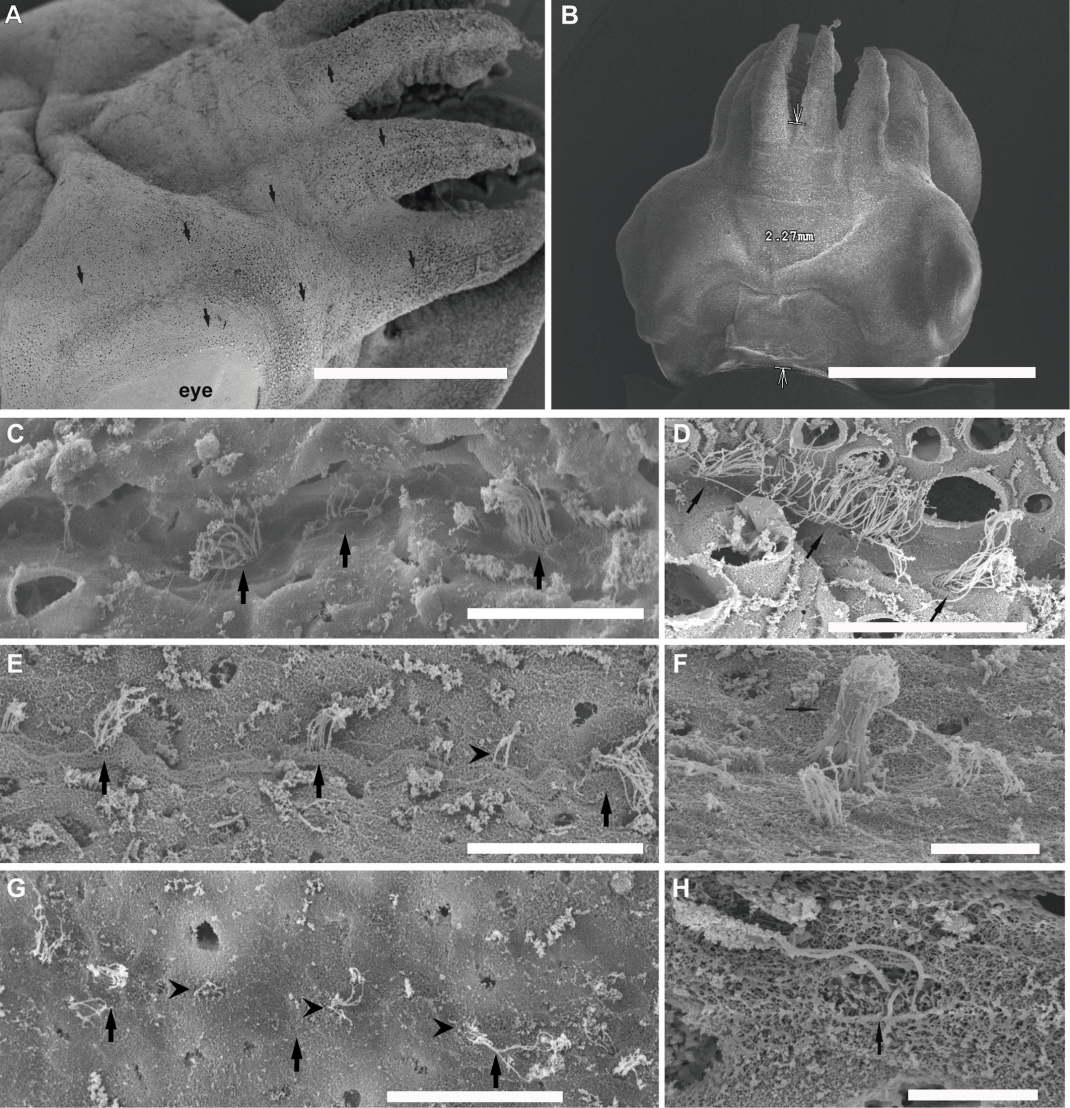
**SEM. *S. officinalis* epidermal lines**. Control animals (A–D). Animals euthanised immediately (E,F) and at 24 h (G,H) after sound exposure. (A) Arrows show lateral lines on three arms and above the eye (L1–L3). (B) Arrows show the length of the lateral line L1 used for the quantification of injuries. (C) Healthy lateral Line L1. Arrows point to the hair cells. (D) Detail from the kinocilia on epidermal lines hair cells. (E) Arrows indicate L1. Hair cells present missing kinocilia (arrowhead). (F) A hair cell exhibits fused kinocilia. (G) Hair cell has almost totally lost its kinocilia (arrows) and the rest of their roots are visible (arrowheads). (H) The hair cells have lost a number of their kinocilia (arrows) and the remaining are fused or bent and flaccid. Scale bars: A=1 mm. B=2 mm. D=30 µm. C,E,H=25 µm. F=10 µm. G=5 µm.

As shown in the literature, *S. officinalis* hatchlings display eight epidermal lines on their head, two dorsally- and two laterally-positioned lines, which continue on the arms. There are an additional pair of L5 short lines on the ventral side of the head and a band of ciliated cells on the ventral funnel surface (Fig. 1).

The whole body of *L. vulgaris* hatchlings presented a variety of hair structures, many of them related to sensory function (Fig. S2). In addition to the epidermal lines (Fig. S2A–E), the animals exhibited rows of hair cell lines covering the mantle (Fig. S2H–J). Additional highly developed structures (olfactory organs) were visible on these individuals (Fig. S2F,G).

**Fig. 2.**
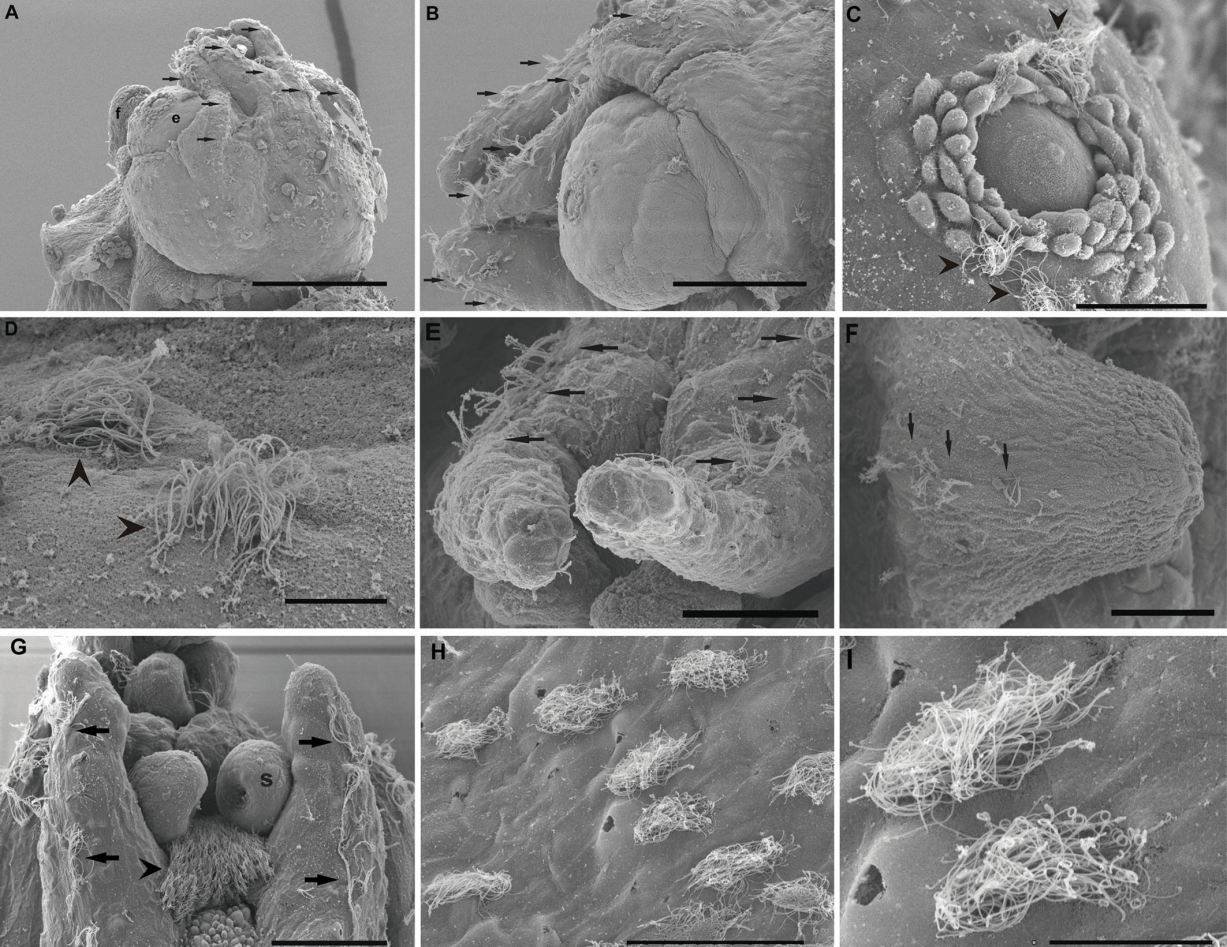
**SEM. *I.coindetii* epidermal lines and accessory ciliated structures.** Control animals. (A) Arrows show the four lines on the dorsal side of the head. e, eye; f, funnel. (B) Detail from A, arrows indicate the highly dense epidermal lines. (C) Bundles of kinocilia (arrowheads) of the Line 3 running above the eye are visible (dorsal side). (D) Detail from C. Note the high density of the kinocilia on the hair cells (arrowheads). (E) Arrows indicate the two Lines 5 on the ventral side. (F) Unique line of the funnel located along its midline. Arrows indicate the lateral line of the funnel. (G) The epidermal lines on the arms are visible (arrows). The arrowhead points to the lip chemoreceptors (s:sucker). (H) Bundles of hair cells covering all mantle surface. (I) Detail of H, it shows the highly developed bundles of hair cells. Scale bars: A=200 µm. B=100 µm. C,E,F,G,H=50 µm. I=20 µm. D=10 µm.

This study shows the first published images of the epidermal lines of *I. coindetii* paralarvae (Fig. 2). The epidermal lines, which present the same distribution as other decapodiforme species [Fig. S1B (Lenz et al., 1995), i.e. five pairs of bilaterally symmetrical lines in the head and arms, and an additional unique line on the ventral funnel surface], are highly dense and reveal different kinocilia distribution and density on the hair cells (Fig. 2A–G).

As in *L. vulgaris* hatchlings, the animals exhibited hair structures covering the whole mantle (Fig. 2H,I). In this case, the hair cells are distributed in bundles over the whole surface (Fig. 2H,I). Additional highly developed structures (lip chemoreceptors) were visible on these individuals (Fig. 2G).

#### The effects of sound on the epidermal line sensory epithelium

**Fig. 3.**
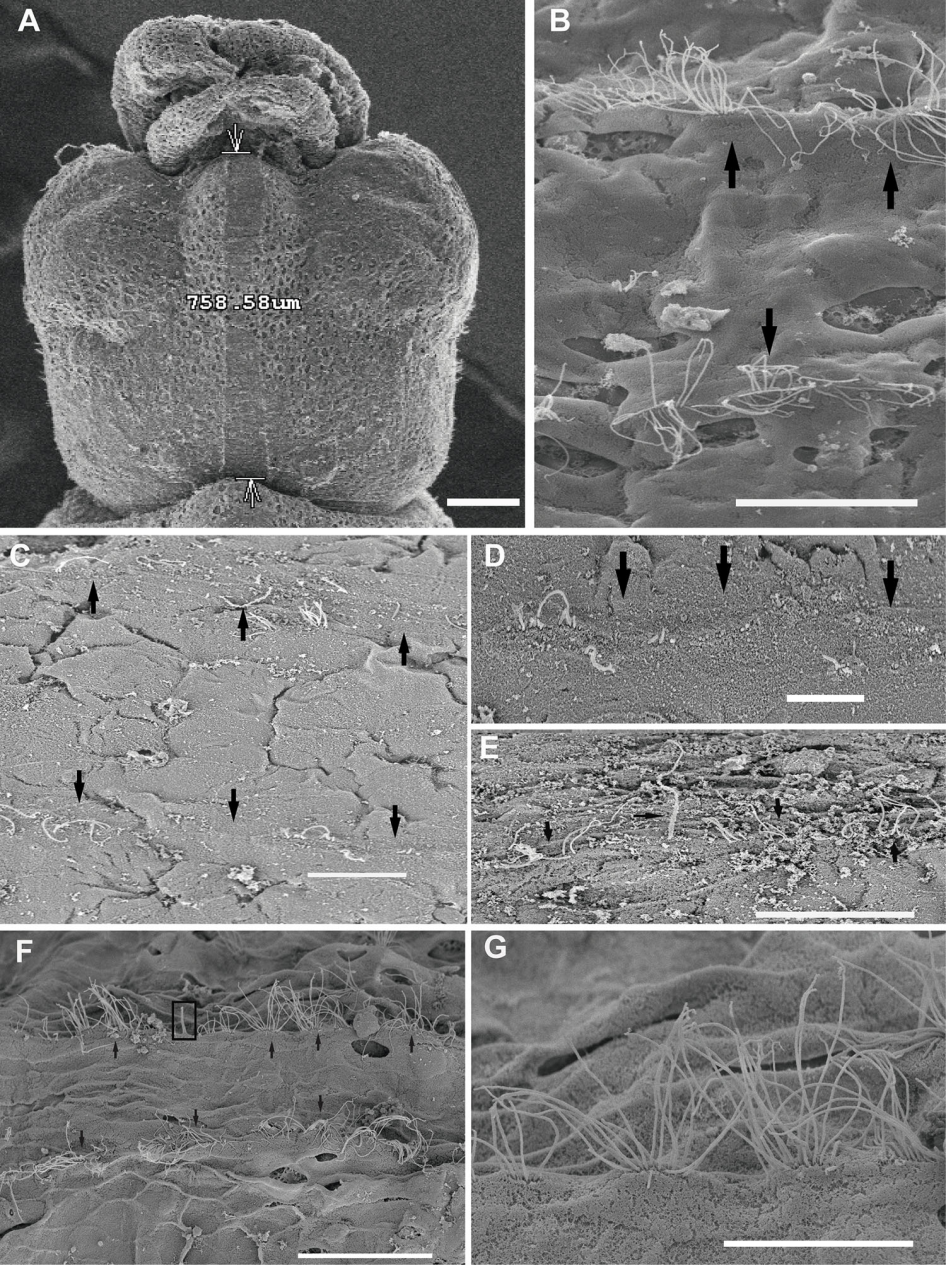
**SEM. *L. vulgaris* epidermal lines.** Control animals (A,B). Animals euthanised immediately (C–E) and at 24 h (F–G) after sound exposure. (A) Dorsal view. Arrows show the length of the lateral line L1 used for the quantification of injuries. (B) Arrows show a detailed view from the pair of lines L1. Note the regular arrangements of the kinocilia hair cells on control animals. (C-D) On L1 some hair cells have almost totally lost their kinocilia (arrows). (E) The remaining kinocilia are bent and flaccid. (F) The two L1 lines (arrows) show a healthy appearance with upright kinocilia of the hair cells arrangements. Some kinocilia can be seen to be fused (black box). (G) Detail from F. Hair cells' kinocilia of L1. Scale bars: A=100 µm. B=25 µm. C=15 µm. D=10 µm. E,G=20 µm. F=50 µm.

**Fig. 4.**
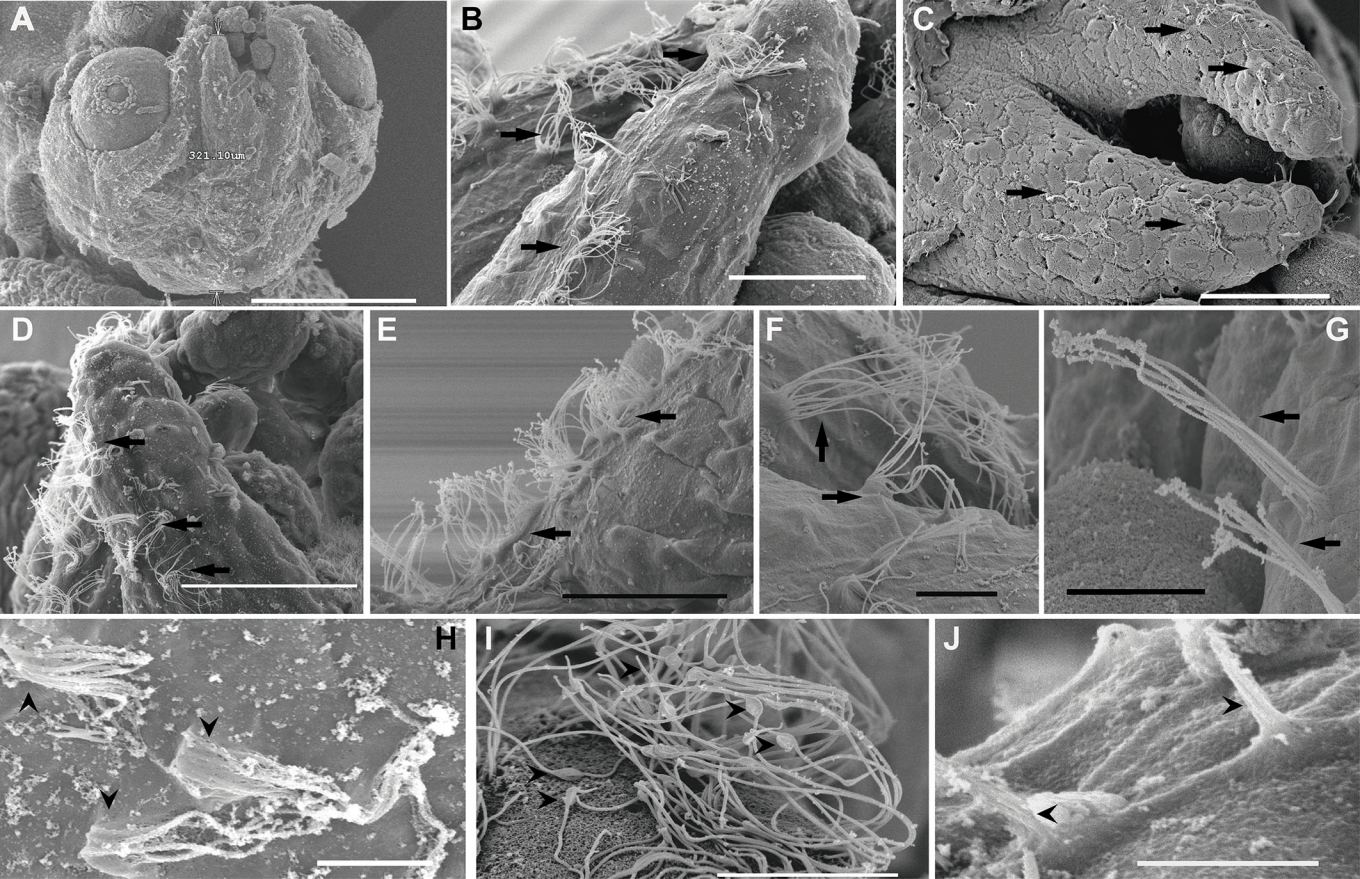
**SEM. *I. coindetii* epidermal lines.** Control animals (A,B,E). Animals euthanised immediately (C,H–J) and at 24 h (D,F,G) after sound exposure. (A) Dorsal view. Arrows show the length of the lateral line L1 used for the quantification of injuries. (B) Healthy hair cell on the lateral line L1 (arrows). (C) Lateral line L1 has almost lost all hair cells. The remaining kinocilia are bent and flaccid. (D) Healthy aspect of the bundles of hair cells on L1. Arrows indicate the bundles of hair cells on the lateral line L1. (E) L1 line shows a healthy appearance with upright kinocilia of the hair cells arrangements. (F–G) Two different views of the kinocilia distribution on the epidermal lines hair cells. (H) Detail of damaged bundle showing the kinocilia bend, flaccid and fused on the basal part. Arrowheads indicate the kinocilia bend, flaccid and fused on the base. (I) Some of the kinocilia on the bundles show blebs (arrowheads). (J) The remaining kinocilia of damaged hair cells are fused (arrowheads). Scale bars: A=200 µm. B,E=30 µm. C,D=50 µm. F,G,I=10 µm. H,J=5 µm.

Just after sound exposure (Figs 1E–F; 3C–E; 4C,H–J), in comparison with the same tissues from control animals (Figs 1A–D; 3A,B; 4A,B,E), damage was systematically observed on the epidermal lines by SEM analysis. Zones previously occupied by hair cells exhihited damage in the epithelium. Some hair cells had dramatically lost almost all kinocilia and the remaining kinocilia were bent and flaccid or fused (Figs 1E–F; 3C–E; 4C,H,J). In some cases the kinocilia on the bundles show blebs as a consequence of sound exposure (Fig. 4I).

In *S. officinalis* hatchlings euthanised at 24 h, the same lesions described above were found with an increase in the severity of expression (Fig. 1G–H). In most samples from *L. vulgaris* and *I. coindetii* hatchlings euthanised 24 h after sound exposure (80% and 90% respectively) (Figs 3F,G; 4D,F,G), the epidermal lines showed a healthy appearance with upright kinocilia on the hair cells arrangements, although some hair cells exhibited fused kinocilia (Fig. 3F).

### Structural and ultrastructural analysis of the statocyst sensory epithelium

As described in adult animals, hatchlings of *S. officinalis* present two statocyst inner sensory systems. The macula-statolith system (gravity receptor system) of the two statocyst cavities is divided into three subunits [macula statica princeps (msp), superior macula neglecta (smn) and inferior macula neglecta (imn)] (Fig. S3). Equally, the crista-cupula system (angular acceleration receptor) shows four rows of different sizes of sensory hair cells (Fig. S3H,I).

The largest unit (msp) of the macula-statolith system is visible in *L. vulgaris* hatchlings (Fig. S4). The crista-cupula system (angular acceleration receptor) is also composed of four rows of different hair-cell types (Fig. S4H,I).

**Fig. 5.**
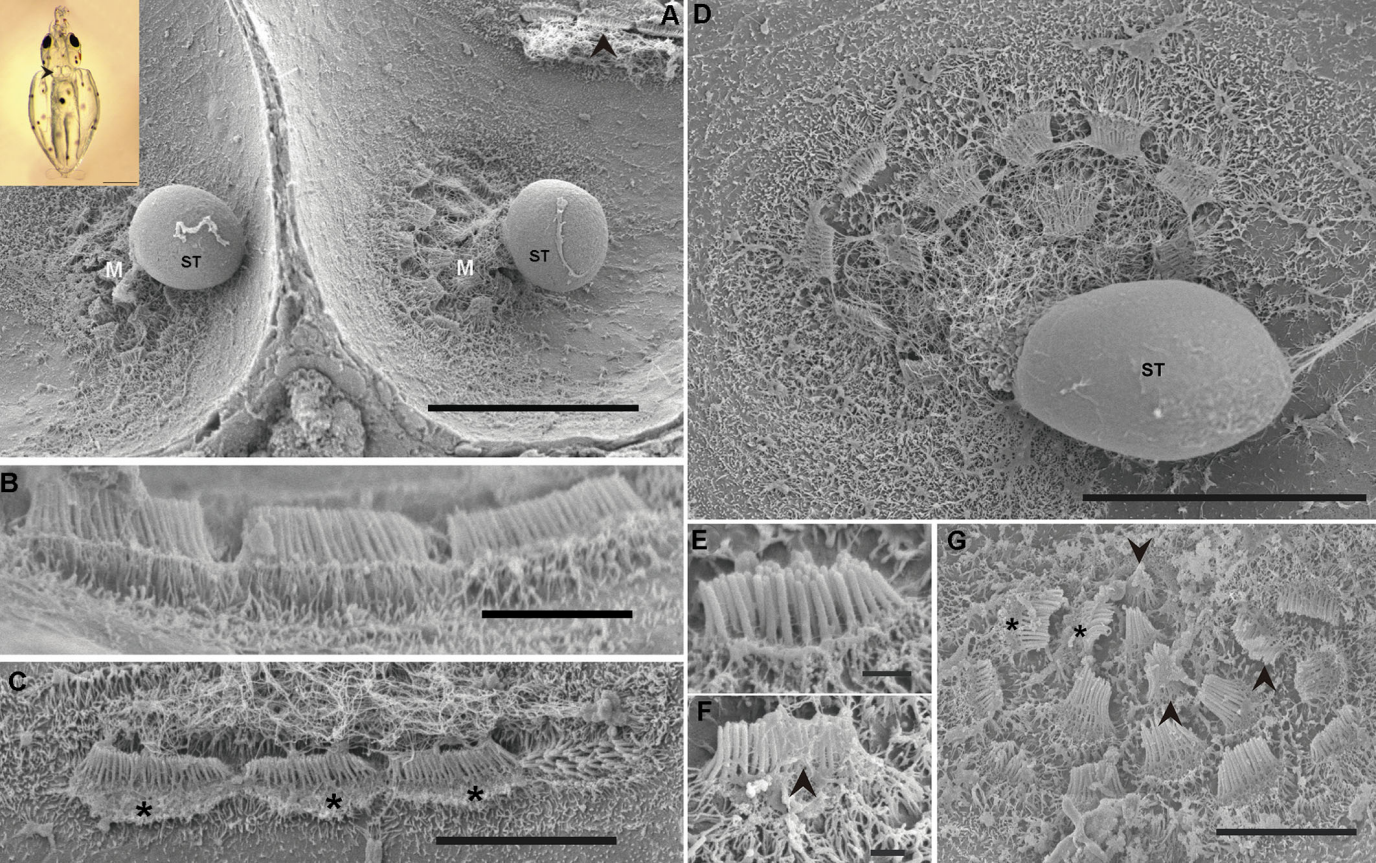
**SEM. *I. coindetii* hatchling inner statocyst morphology.** Inset in A: LM. Photomicrograph of *I. coindetii* hatchling. Control hatchlings (A,B,D,E), euthanised immediately (F) and 24 h after sound exposure (C,G). Inset in A: upper view of hatchling broadtail squid (*I. coindetii*) from *in vitro*, kindly provided by Dr Roger Villanueva (Villanueva et al., 2011). Arrowhead shows the position of the statocyst on the posterior-ventral position of the head. The statoliths are visible in the statocysts. (A,D) The statocyst cavities have been opened transversally. Each statocyst cavity shows the statolith (ST) attached to the macula statica princeps (M). Note the hair cell kinocilliary groups are arranged in nearly concentric rings around a centre. Arrowheads point to the only segment of crista visible at this stage, covered by some rests of cupula. (B) Crista system. In this case only one row of sensory hair cells is visible*.* (C) On an exposed hatchling, the crista present the apical pole of hair cells partially extruded (asterisks). (E) Detail of a hair cell. Note the healthy aspect of the bundle of kinociliary group. (F) The hair cell shows the kinocilia have fused immediately after sound exposure (arrowhead). (G) Some hair cells present the apical pole extruded from the sensory epithelium (black asterisks). Arrowheads point to disorganized, bent or fused kinocilia. Scale bars: Inset in A=0.5 mm. A=30 µm. B=5 µm. C,D=20 µm, G=10 µm, E,F=1 µm.

This study shows the first published images of statocyst inner morphology in *I. coindetii* paralarvae (Fig. 5). Here, as opposed to adult animals, only the largest unit (msp) of the macula-statolith system is present in *L. vulgaris* hatchlings (Fig. 5A,D). The epithelium of the crista-cupula system shows only one row of hair cells, versus the normal distribution of four rows normally seen in adult specimens (Fig. 5B,C).

#### The effect of sound on the statocyst sensory epithelium

In all samples of *S. officinalis* and *I. coindetii* hatchlings, crista epithelium showed obvious signs of damage, including bent kinocilia and cellular material extrusion after exposure to sound. The epithelium was fractured in-between some of the four rows that integrate the crista epithelium. Some hair cells had their apical poles partially, or totally extruded (Fig. S3J; Fig. 5C). No lesions were detected in the smaller subunits of the macula-statolith system (smn and imn, Fig. S3D–G) in *S. officinalis*.

**Fig. 6.**
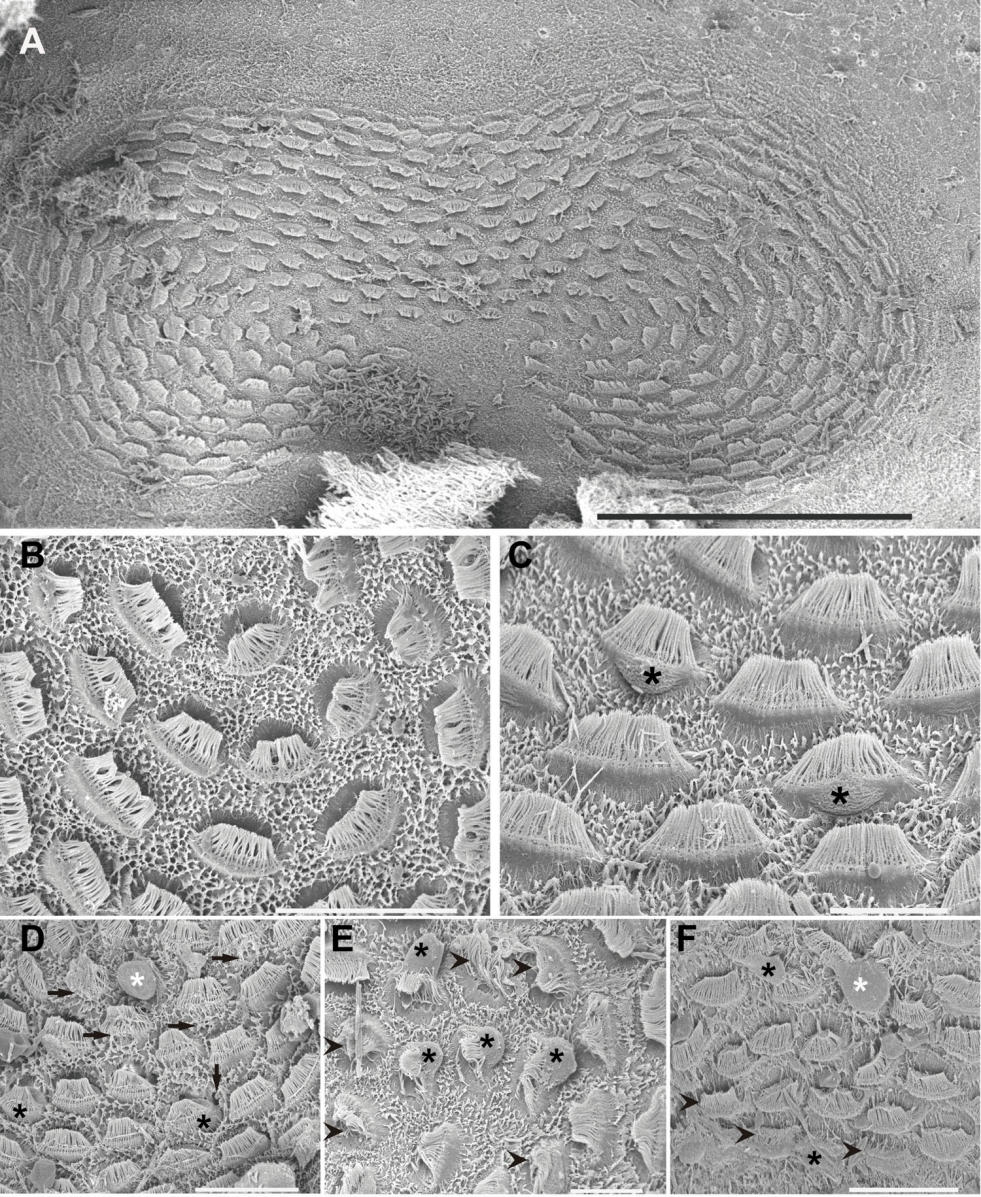
**SEM. *S. officinalis* macula statica princeps**. Control animals (A,B), euthanised immediately (C) and 24 h after sound exposure (D–F). (A) The largest subunit (msp) of the macula-statolith system is shown. Some statolith fragments are visible in the low part of the image. (B) Detail from A. Note the arrangements of the kinociliary groups of the hair cells in regular lines following the epithelium shape. (C) Two hair cells show the beginning of the extrusion of the apical pole above the epithelium into the statocyst cavity (asterisk). (D) Some hair cells present the cell body ejected from a large region of the sensory epithelium (black asterisks). Most of the hair cells show holes in the base caused by the extrusion of inner material (arrows). Note the cellular material extruding (white asterisk). (E) The cell bodies of most of the hair cells are protruding into the statocyst cavity (asterisk). On other cells, the kinocilia are bent, disorganized or fused (arrowheads). (F) Some hair cells present the cell body ejected (black asterisks). In one cell the cellular material extruding is visible (white asterisk). Arrowheads point to the fused kinocilia. Scale bars: A=100 µm. B,D,F=20 µm. C,E=10 µm.

**Fig. 7.**
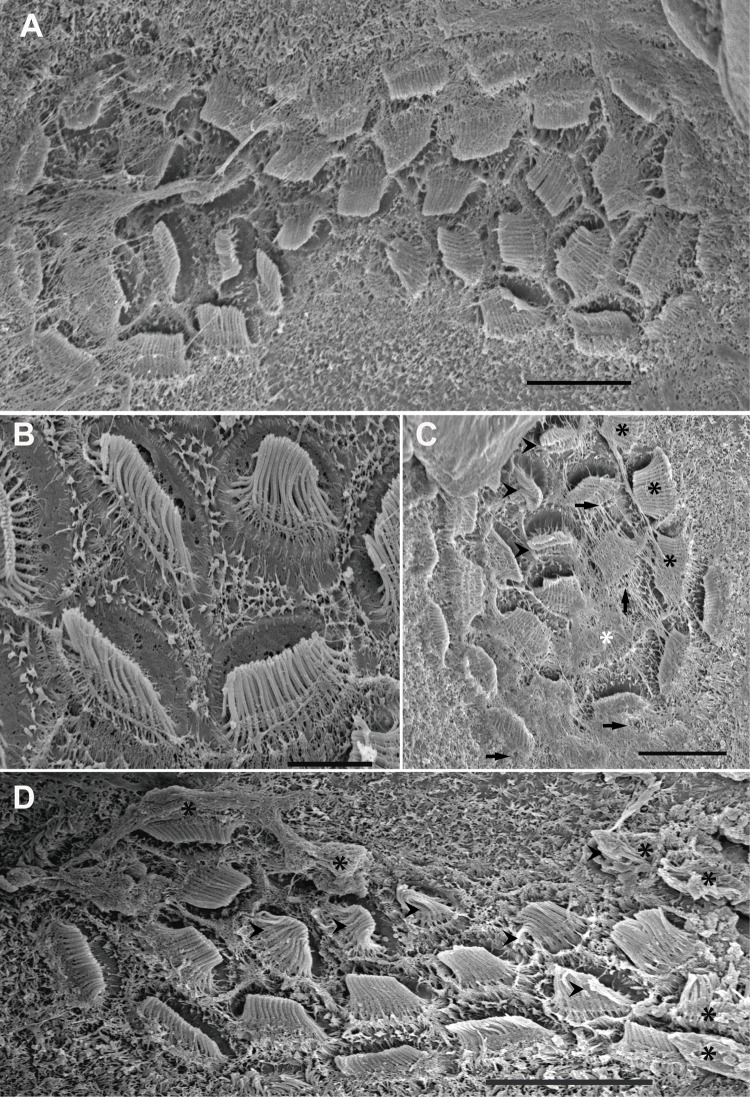
**SEM. *L. vulgaris* statocyst macula statica princeps**. Control animals (A,B), euthanised immediately (C) and 24 h after sound exposure (D). (A) Upper view of *msp*. Note the arrangements of the hair cells kinociliary groups. (B) Detail from A. Note the space under the hair cell which permits the polarized movement of each kinocilliary group of a hair cell. (C) Some hair cells present the apical pole extruded from the sensory epithelium (black asterisks). Other hair cells show holes in the base caused by the extrusion of inner material (arrows). Note the space left by an extruded hair cell (white asterisk). Arrowheads point to disorganized, bent or fused kinocilia. (D) The cell bodies of some hair cells are protruding into the statocyst cavity (asterisk). On other cells, the kinocilia are bent, disorganized or fused (arrowheads). Scale bars: A,B=5 µm. C,D=10 µm.

In comparison with the same tissues from control animals (Figs 6A–B; 7A–B; 5A,D,E), damage was systematically observed in the msp by SEM analysis, just after sound exposure (Figs 6C, 7C, 5F). Some hair cells had totally lost their kinocilia or showed bent, flaccid or fused kinocilia and a large number of hair cells had their apical pole extruded above the sensory epithelium into the statocyst cavity, and the expulsion of the cellular material left holes in the base of hair cells (Figs 6C, 7C, 5F).

In animals euthanised 24 h after sound exposure (Figs 6D–F, 7D, 5G) the same lesions described above were found with an increase in their severity.

See Table S1 for a summary description of the observed effects on the different sensory systems and species.

### Quantification and data analysis

**Fig. 8.**
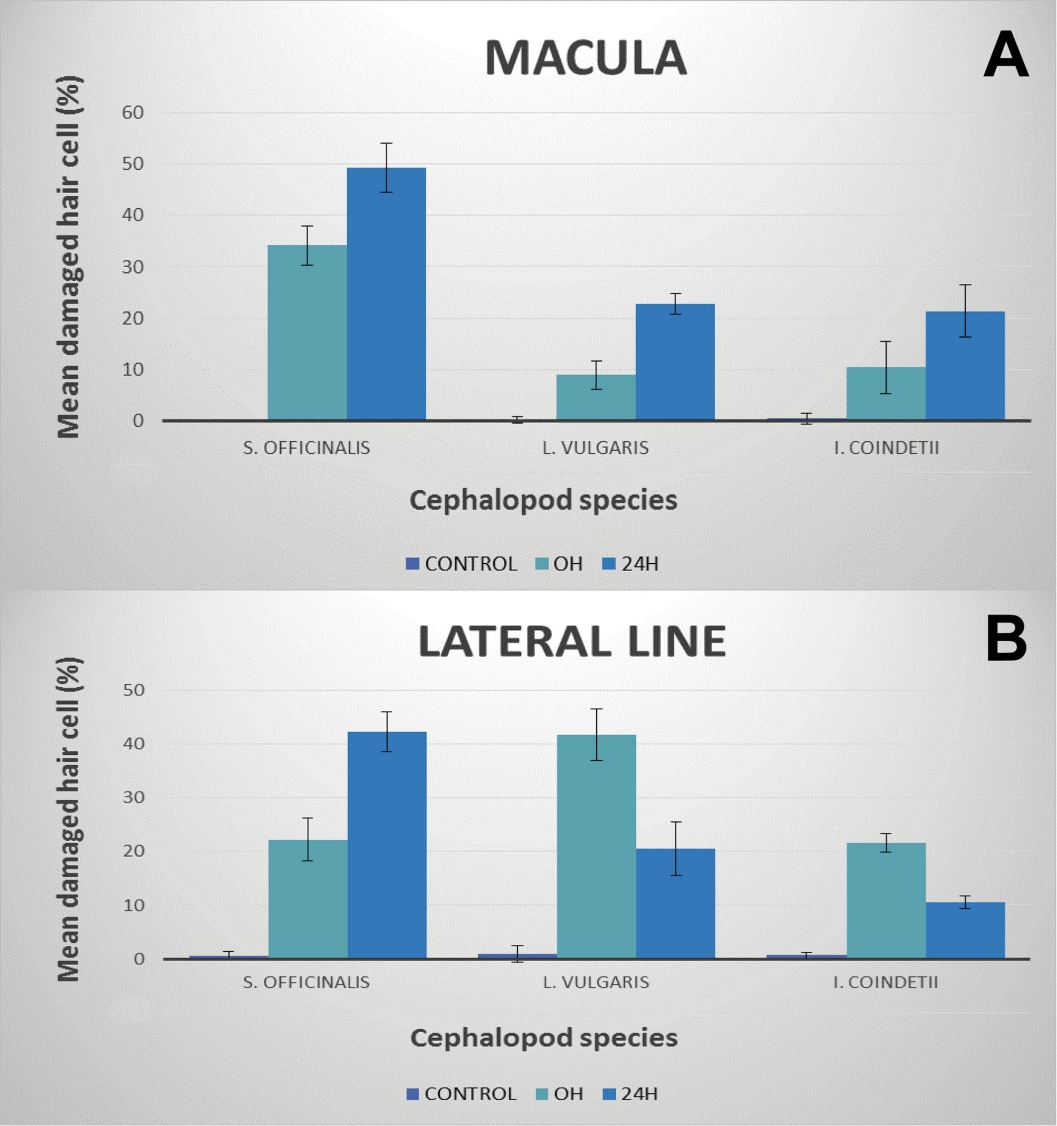
**Statistical analysis of the damage.** (A) Mean (±s.e.) damaged hair cell on the whole sensory area of the statocyst macula versus time. Note the increase of damaged hair cells versus controls with increase of time. Each bar is the average over whole area of macula with the line indicating the standard deviation. The percentage was computed by dividing with the total count for each individual sample. [*S. officinalis* and *L. vulgaris* (*n*=40) and *I. coindetii* (*n*=20)]. (B) Mean (±s.e.) damaged hair cell on the lateral line sensory epithelium versus time. Note the increase in *S. officinalis* and the decrease in *L. vulgaris* and *I. coindetii* hatchlings of damaged hair cells versus controls with increase of time. Each bar is the average over area of lateral line L1 with the line indicating the standard deviation. The percentage was computed by dividing with the total count for each individual sample. [*S. officinalis* and *L. vulgaris* (*n*=40) and *I. coindetii* (*n*=20)].

In the three species, the number of damaged cells (extruded and missing hair cells) increased with time in the statocyst msp sensory epithelium (Fig. 8A). The presence of extruded cells showed the start of severe damage after sound exposure. In the lateral line sensory epithelium, the damage increased with time in the case of *S. officinalis* hatchlings; however, in the other two species, after the initial increase in the lesions in comparison with control animals, the severity of the lesions decreased with time (Fig. 8B). Their severity was quantified as a number of damaged cells compared to control specimens. The deviation in damage was tested (Wilcoxon rank sum test) in control animals at 0 h and twice at 24 h, as well as recovery if the damage at 0 h was higher than at 24 h. The *P* values were all either very close to 0 or to 1 and are given below.

### Test 1: difference between control and exposed individuals

In all cases, the severity of the lesion between control animals and exposed individuals observed at 0 h differed significantly for both epithelia (the medians were not equal to the median of the macula/lateral line control at 0 h, *P*<0.001). We conclude that statocyst and lateral line sensory epithelia were affected by exposure to sound in the three species.

### Test 2: difference in numbers between individuals observed at 0 h and 24 h after exposure

The quantification analysis showed that the severity of the lesions, which were quantified as the number of extruded/missing hair cells, increased with time for the three species (Fig. 8A). In all cases, after performing statistical tests, the lesions in the msp after 24 h were not significantly less than at 0 h (the macula did not show more damage at 0 h, *P*=1.000).

Regarding the differences in the lateral line sensory epithelia, we obtained different results depending on the species. In the case of *S. officinalis* the lesion number in the lateral line after 24 h was not significantly lower than at 0 h (the lateral line did not show more damage at 0 h, *P*=1.000). In the cases of *L. vulgaris* and *I. coindetii*, the lesion number in the lateral line after 24 h was significantly lower than at 0 h (*P*<0.001). For these cases, the lesion number at 24 h was tested against the control and was found to be significantly different (the median of the lateral line control was not found to be equal, *P*<0.001). We conclude that in *S. officinalis* hatchlings there was an increase in the severity of the lesions with time. The other two species, *L. vulgaris* and *I. coindetii*, presented a decrease in the severity of the lesions with time in the lateral line sensory epithelium. Despite this decrease in the severity of the lesions 24 h after sound exposure, there were still significant differences in the number of lesions compared with the control animals (*P*<0.001).

## DISCUSSION

### Description of hatchling sensory structures

Literature on cephalopod hatchlings and embryonic stadia sensory systems, including statocyst and lateral line, is scarce [see as a review, Villanueva and Norman (2008)]. The neural basis of the ability of some coleoid cephalopods’ embryonic stadia to respond to a variety of sensory stimuli during early development in the egg capsule was assessed by the analysis of the emergence sensory structures within the developing epidermis (Buresi et al., 2014). Lateral lines and statocyst structures seem to be present from the initial stadia of the developmental process (Buresi et al., 2014), highlighting their fundamental role in the animal’s survival.

In addition, some typical ciliated structures that are not linked to sound perception (e.g. chemoreceptors) such as the olfactory organ with high cilia density that is located near the funnel in *Loligo* paralarvae, were visible on all groups of samples. No lesions were visible there. The early differentiation of the olfactory organ (Fig. S2F,G) in *L. vulgaris* hatchlings and the peculiar development of the epidermis together with its sensory cells allow comparing developmental processes within the molluscs phylum (Buresi et al., 2014; Polese et al., 2016). These results confirm that the statocyst and the lateral line sensory epithelia are specialised in sound perception, and thus can suffer acoustic trauma when exposed to loud sound sources while the other observed sensory epithelia are not affected.

The distribution of epidermal lines in *I. coindetii* paralarvae is very similar to other described decapodiforme species such as *Sepiola affinis* [Fig. S1B (Lenz et al., 1995)], *S. officinalis* and *L. vulgaris* (five pairs of bilaterally symmetrical lines in head and arms, and an additional unique line on the ventral funnel surface).The epidermal lines present different kinocilia distribution and density in the hair cells. The animals exhibited bundles of hair cells covering the mantle surface.

We showed in *I. coindetii*, additional sensory ciliated structures like lip chemoreceptors (Fig. 2); ciliated receptors and sensory cells are also described on the finger-like papillae that distally fold the muscular lip around the beaks in *O. joubini* (Emery, 1975). Although their presence in octopus paralarvae has not yet been described (Villanueva and Norman, 2008) we were able to identify them in *I. coindetii* (Fig. 2).

On the other hand, the inner sensory epithelia of the *I. coindetii* statocyst was also shown for the first time (Fig. 5). In contrast with *S. officinalis* (Fig. S3) and *L. vulgaris* (Fig. S4) hatchlings that exhibit all the inner statocyst sensory structures present on adult individuals, *I. coindetii* shows only the largest unit of the macula–statolith system (msp) and a short row of hair cells on the crista epithelium. Compared to the other two hatchling species studied here, which – as is found in adults – present four rows of different types of hair cells on the crista epithelium, in *I. coindetii* paralarvae statocyst, only one row of scarce, sensory hair cells is visible (Fig. 5B,C). This feature is probably due to the fact that the hatchlings of *I. coindetii* are still in early larval stages and will evolve a greater number of more complex structures later in their development.

### Effects of sound on hatchling statocyst

In previous studies, it was shown that the exposure to sound resulted in permanent and substantial alterations of the sensory epithelia of the statocysts. This morphological and ultrastructural evidence of massive acoustic trauma was assessed in adult individuals of four cephalopod species subjected to low-frequency controlled-exposure experiments in laboratory (André et al., 2011; Solé et al., 2013a,b) and offshore (Solé et al., 2017) conditions. Few studies summarize the effects of the external environmental events on statocyst of cephalopod hatchlings (Zumholz et al., 2007; Colmers et al., 1984; Kaplan et al., 2013). Some results (Colmers et al., 1984; Kaplan et al., 2013) reported behavioural effects characterized by the inability to control orientation while swimming, caused by the abnormalities of the statocyst neuroepithelial suprastructures (absence or malformation of statolith or cupula) as a consequence of environmental causes (temperature and chemical composition of the sea water, rearing systems, genetic causes, ocean acidification) (Colmers et al., 1984; Villanueva, 2000; Hanlon et al., 1989; Zumholz et al., 2007; Kaplan et al., 2013). This study provides the first analysis of the effects of sound on inner epithelia of statocyst and lateral line structures in Cephalopod hatchlings.

In the hatchling cephalopod species, lesions were described in the macula statica princeps and crista, both statocyst inner sensory epithelia. The effects of sound exposure on the statocyst epithelia summarized here are similar to previous results (André et al., 2011; et al., 2013a,b, 2017) in adult cephalopod specimens: bending, flaccid fused or lost kinocilia, cellular material extrusion, some hair cells had their apical poles partially or totally extruded above the sensory epithelium. However, these lesions presented a different incremental severity over time, since in hatchlings we quantified a similar effect as found in adults after 48 h, almost immediately after exposure. Regenerative processes potentially due to cell division and/or differentiation were not observed, similarly to what was found in adult cephalopod statocyst sensory epithelia euthanised after 96 h after sound exposure (Solé et al., 2013a).

### Effect of sound on hatchling lateral lines

Although fish lateral lines are shown to act as displacement detectors and are potentially stimulated by particle motion components of sound sources (Higgs and Radford, 2013), the capacity of cephalopods to sense local water movements is still under discussion. In 1928, Naef (1928) first mentioned 4–5 pairs of regularly arranged lines of epidermal cells on the head and on the arms of embryonic cephalopods. Sundermann (1983) described these cells as ciliated sensory cells and Budelmann et al. (1991) demonstrated that these lines of cells served to detect small water movements. These analogue lateral lines are involved in prey and predator detection.

While the controls showed no lesions in the epidermal lines, all the exposed individuals displayed acoustic trauma, but its evolution over time was different depending on the species. In *L. vulgaris* and *I. coindetii* paralarvae, the damage was observed in the epidermal lines immediately after exposure. A high number of hair cells had lost kinocilia or presented fused, bent and flaccid kinocilia. But, in contrast with what was observed in statocyst structures, in the paralarvae epidermal lines lesions were not gradually more pronounced after 24 h. Surprisingly, some randomly distributed areas of the epidermal lines of the exposed animals showed high cell density. We could only find fused kinocilia that remained in some hair cells even 24 h after sound exposure.

In *S. officinalis* juveniles, damage was also observed in the epidermal lines immediately after exposure. A high number of hair cells had lost kinocilia or presented fused, bent and flaccid kinocilia and the lesions in the juveniles epidermal lines were gradually more pronounced in individuals after 24 h as in the case of the statocyst lesions. The different evolution in *S. officinalis* juveniles and *L. vulgaris* and *I. coindetii* paralarvae could be due to the different sizes of hatchlings from the three species, which would correspond to different live stages and strategies. The larger size of cuttlefish hatchlings is consistent with the fact that cuttlefish become a benthic species immediately after hatching, whereas the other two species present a planktonic phase before they sufficiently grow to become juveniles. These differences may involve changes in the typology of the lesions produced by noise impact due to variations in sound perception in different water layers.

In the avian cochlea and vestibular organs, and in the vestibular organ of mammals, post-traumatic hair cell regeneration was shown to occur after either acoustic trauma or drug poisoning, when apoptosis is not involved (Corwin and Cotanche, 1988). Several mechanisms are believed to be involved in such regeneration, including proliferation followed by differentiation of non-sensory epithelial cells, direct transdifferentiation of supporting cells into hair cells, and reparation of damaged hair cells. In the same way, fish are capable of regenerating sensory hair cells in the inner ear after acoustic trauma (Schuck and Smith, 2009). Mechanosensory hair cells within zebrafish larvae and other fish species' lateral lines spontaneously regenerate after being damaged or destroyed by acoustic or ototoxic exposure (Ma et al., 2008; Mackenzie and Raible, 2012; Monroe et al., 2015). These processes generally occur within one to several weeks after the hair cells’ death. In the present study, the survival time of individuals was no more than 24 h following the sound exposure, which is too short a period of time to observe possible regenerative processes due to cell proliferation. This survival time was not related to the exposure to sound, but to the food reserve limit that the hatchling receives from its vitelline sac. The present results cannot state, therefore, whether the exposure to sound induced permanent lesions in the exposed individuals.

These results constitute a basis for future research to determine whether some of the epidermal lines are more sensitive than others to sound, and this could also be linked to the mechano-reception role of these sensory organs versus their sensitivity to acoustic pressure.

### Critical periods of susceptibility to acoustic trauma in cephalopod hatchlings

Previous experimental studies on mammals (Lenoir et al., 1979, 1986; Uziel, 1985) and birds (Cousillas and Rebillard, 1985) demonstrated critical periods of increased susceptibility to acoustic trauma in young animals. During such critical periods, the same sound exposure appears to be more traumatic in young animals than in adults (Falk et al., 1974; Price, 1976; Jensen et al., 2015). These critical periods generally coincide with the last stages of cochlear anatomical development, when the organ has just acquired its adult functional properties level (Lenoir et al., 1979; Uziel, 1985; Lenoir and Pujol, 1980). During such periods of increased sensitivity to sound, noise exposure can damage the cochlea at physiological and histological levels (Lenoir et al., 1979, 1986; Lenoir and Pujol, 1980).

The anatomical damage in the hair cells of young avian basilar papilla is similar to those classically described in the literature regarding adult birds (Saunders and Tilney, 1982) – absence of cilia, disorganization of their structure, reduction in size of cuticular plates, enlargement of supporting cells, presence of sensory cells with giant cuticular plate, growth of microvilli on the cuticular plate – and outer hair cells in the cochlea of mammals (Hunter-Duvar, 1977; Robertson and Johnstone, 1980). In chicks, the developmental change seems to end at post-natal day 1, which also corresponds to the end of the anatomical and functional maturation of the basilar papilla (Cousillas and Rebillard, 1985).

The present results of the lateral line and statocyst of cephalopod hatchlings showed similar anatomical effects after sound exposure. All exposed cephalopod hatchlings displayed acoustic trauma, but its evolution over time was different depending on the species. *L. vulgaris* and *I. coindetii* paralarvae presented a high number of hair cells that had lost kinocilia or presented fused, bent and flaccid kinocilia in lateral line and statocyst immediately after sound exposure. While statocyst anatomy showed an increase in damage with time, the lateral line did not follow the same pattern 24 h after sound exposure. According to the literature on mammals and birds, we can hypothesize a similar critical period of susceptibility to acoustic trauma for the *L. vulgaris* and *I. coindetii* paralarvae lateral lines that would not continue after hatching. But, as in these two species, the lesions in the statocyst structures were more pronounced after 24 h, thus we can suppose that the critical period of susceptibility to sound does not coincide with time. These results point to a major susceptibility of the statocyst, possibly due to a later completion of the anatomical differentiation of the statocyst structures, consistent with its greater structural complexity.

In *S. officinalis* juveniles, statocyst and lateral line structural damage was observed immediately and the lesions were gradually more pronounced 24 h after sound exposure. The larger size of cuttlefish hatchlings and their benthic stage would indicate a higher level of development in this species, which would be consistent with their potentially complete anatomical differentiation during the embryonic phase, prior to hatching. This could be responsible for the different evolution in the anatomical damage that is more consistent with the evolution of the effects on sensory epithelia in adults.

### Conclusion

Our study devised a novel approach to quantify and compare damage in the sensory systems responsible for acoustic perception (statocyst and lateral lines) in cephalopod hatchlings, by examining hair cells, which enabled meaningful comparison across a broad number of samples. In particular, our estimate of the proportional damage to the whole hair-cell surface provided a general idea as to the mechanoreceptor’s sensitivity and allowed the first qualitative and quantitative analysis of sound-exposure damaged sensory epithelia in cephalopod hatchlings. Our results indicate an increase in the severity of the lesions in the statocyst sensory epithelia with time, common to all species. In hatchlings the severity of these lesions was shown to increase much faster than in adults, but due to the limited survival time of the studied individuals (related to the vitelline sac reserve) we could not properly assess the consequence of these lesions with their capacity to grow over time. Nevertheless, if we relate this acoustic trauma to what was observed in adults, who stopped eating and breeding after sound exposure, it is likely that hatchlings exposed to noise could not eat properly and would eventually die.

On the other hand, the different evolution of the sound exposure damage in the lateral line of *S. officinalis* juveniles and *L. vulgaris* and *I. coindetii* paralarvae could be explained by the different life styles and size of hatchlings, corresponding to different life stages. In addition, this difference in damage evolution could be associated with critical periods of enhanced sensitivity for acoustic trauma; linked to the end of the anatomical and functional maturation of their sensory structures, as reported in the mammalian cochlea and avian basilar papilla. It is therefore essential to better understand the sensory-structure development phases in these species to link them to possible critical periods of supranormal susceptibility to acoustic trauma.

## MATERIAL AND METHODS

### Cephalopod individuals

Hatchlings of *S. officinalis*, *L. vulgaris* and *I. coindetii* were used in this study. *S. officinalis* and *L. vulgaris* eggs were obtained by local fishermen from the Catalan coast (north-west Mediterranean Sea). *I. coindetii* hatchlings were obtained from the Institute of Marine Sciences (CSIC) installations, from experiments described by Villanueva et al. (2011) using *in vitro* fertilization techniques. All hatchlings were kept in a closed system of recirculating natural seawater (at 18–20°C, salinity 35 and natural oxygen pressure). Some of these hatchlings were used as controls and were kept in the same conditions as the experimental animals until we exposed the latter to noise in an independent tank, euthanizing them following the same sequential process as with the sound-exposed group.

### Ethics

The experimental protocol strictly complied with the current ethical and welfare considerations when dealing with cephalopods in scientific experimentation (Moltschaniwskyj et al., 2007; Fiorito et al., 2015). This process was also carefully analysed and approved by the Ethical Committee for Scientific Research of the Technical University of Barcelona, BarcelonaTech (UPC).

### Sound exposure protocol

The eggs were kept in a rearing tank until they hatched, which ranged from a few hours to a few weeks, the protocol then included exposing the experimental individuals to sound. Sequential controlled exposure experiments (CEE) were conducted on hatchlings of *S. officinalis* (*n*=80), *L. vulgaris* (*n*=80) and *I. coindetii* (*n*=40)*.* An additional set of the same number of hatchlings of the same species were used as control and sequentially processed (same procedure as with noise-exposed individuals) right after being hatched, before and after the CEE. The exposure consisted of a 50–400 Hz sinusoidal wave sweeps with 100% duty cycle and a 1 s sweep period for 2 h. The sweep was produced and amplified through an in-air loudspeaker while the level received was measured by a calibrated B&K 8106 hydrophone (RL=157±5 dB re 1 μPa with peak levels up to SPL=175 dB re 1 μPa). The euthanasia process was identical for controls and exposed animals. After the exposure, the individuals not euthanised immediately were placed in a maintenance tank [see tank characteristics in Solé et al. (2013a) and André et al. (2016)]. The independent experimental tank was located in a separate location, acoustically isolated from the maintenance tanks. Following exposure, the samples were obtained from the individuals (exposed and controls) at 0 h and 24 h after sound exposure and were processed for routine scanning electron microscopy (SEM) procedures.

### Imaging techniques

In all experiments, half of the total number of individuals were used for analysing the statocyst and the other half were devoted to the lateral line analysis. For the statocyst analysis, isolated head preparations were obtained by decapitation. The hatchling heads with opened statocyst were fixed for observation and analysis. For fixation, the statocyst cavity was opened and special care was taken to prevent mechanical damage to the inner tissues. For the lateral line analysis, the whole body was fixed and processed according to routine SEM procedures. At this step, some light microscopy images were obtained to clarify the sensorial hatchling sensory structures.

#### Scanning electron microscopy (SEM)

80 statocysts of *S. officinalis*, 80 of *L. vulgaris* and 40 of *I. coindetii* from the following hatchling exposed specimens were used in this study: 40 *S. officinalis* (mantle length 1–1.25 cm), 40 *L. vulgaris* (mantle length 2–3 mm), 20 *I. coindetii* (mantle length 1.3–1.5 mm), in addition to the whole body (the two lateral line L1 were observed) of 40 *S. officinalis* (mantle length 1–1.25 mm), 40 *L vulgaris* (mantle length 2–3 mm), and 20 *I. coindetii* (mantle length 1.3–1.5 mm). One half of the total number of individuals were euthanised at 0 h and the rest at 24 h after sound exposure. The same number of statocysts from exposed and control animals were fixed and processed for the analysis. Fixation was performed in 2.5% glutaraldehyde for 24–48 h at 4°C. Hatchling heads and whole body samples were dehydrated in graded alcohol solutions and critical-point dried with liquid carbon dioxide in a Leica CPD030 unit (Leica Microsystems, Vienna, Austria). The dried samples were mounted on specimen stubs with double-sided tape. The mounted tissues were gold-palladium coated with a Q150R5 sputter coated unit (Quorum Technologies, Ltd., Laughton, UK) and viewed with a variable pressure Hitachi S3500N scanning electron microscope (Hitachi High-Technologies Co., Ltd, Tokyo, Japan) at an accelerating voltage of 5 kV at the Institute of Marine Sciences (ICM) of the Spanish Research Council (CSIC) facilities.

### Quantification and data analysis

We considered, for the quantification of the lesions on lateral lines, the region comprising the sensory area of the pair of lateral lines L1. Between the different lateral lines that compose the system we chose this structure because its dorso-central position allowed us the best visualization of the sensory epithelium.

For the quantification of the inner statocyst epithelia (although we observed the presence of lesions on other inner statocyst epithelia, like crista) we only considered the region including the whole sensory area of the macula statica princeps. This structure was chosen because it represents the largest subunit of the macula-statolith system, and due to its anterior location and relative flat structure, it appeared to be the best to visualise the sensory epithelium. Finally, the generally small size of this structure in cephalopod hatchlings allowed us to quantify lesions on the whole surface of the macula.

Although we observed the presence of abnormal features on the surface of sound-exposed epithelia (e.g. bundle of kinocilia partially or entirely missing, bent or fused) as well as differences in hair cell appearance, hair cell damage was quantified by classifying the hair cells as intact (hair cell undamaged) or extruded/missing (hair cell partially or totally extruded of the epithelium/hole in the epithelium caused by the total extrusion of the hair cell) because these are well-defined categories and easier to compare. The presence of extruded cells determined our threshold of a severe lesion after sound exposure. For all animals, the lesions were assessed for both macula statica princeps and the two lateral L1 lines. The lesions were measured as the number of extruded or missing cells divided by the number of total cells on the inspected surface. The two measurements per system, per animal, were then averaged for statistical tests, resulting in 20 values for the macula and lateral line of the *S. officinalis* and *L. vulgaris* and 10 values for *I. coindetii* (for each system, statocyst and lateral line).

For each area/species/sample we had extruded/missing cell count/area and total cell count/area values. For these two, counts were divided to compute the ratio of missing cells. We had two ratios per animal, these were averaged. We then used rank sum tests (standard Matlab command, either one- or two-tailed depending on testing for difference only, or difference in a specific direction) to test the means. *P*-values were rounded to two decimals.

The data (Table S2) in general did not follow normal distributions (tested with Anderson-Darling) and a non-parametric test (Wilcoxon rank sum withα=0.05) was selected to explore a change in lesions after sound exposure. Two tests were performed, one to test for a difference in lesions between control and exposed animals at 0 h, and one to test changes in the lesions ratio after 24 h.

## Supplementary Material

Supplementary information
