# Supplementary material for: A critical period of susceptibility to sound in the sensory cells of cephalopod hatchlings
Source: Biol Open. 2018 Oct 5;7(10):bio033860. doi: 10.1242/bio.033860 (PMC6215419; doi:10.1242/bio.033860)
Supplement: Supplementary information [file biolopen-7-033860-s1.pdf]

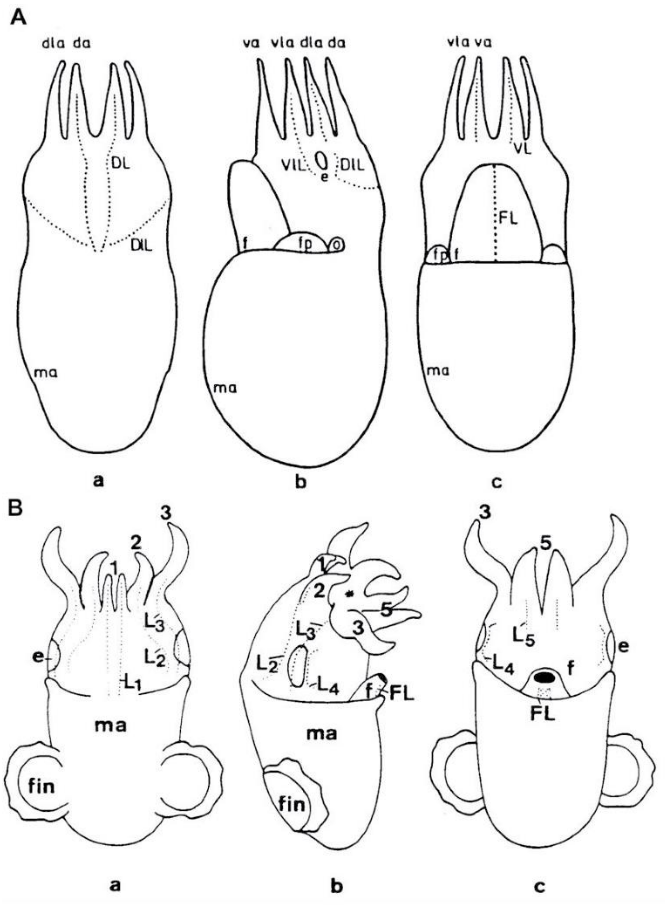

Fig. S1. Schematic drawings, showing the arrangements of the epidermal lines of Octopus vulgaris (A) and Sepia affinis (B) (a: dorsal, b: lateral, c: ventral sight, 1-5: number of arms; da: dorsal arm, DL: dorsal line, dla: dorsolateral arm, DlL: dorsolateral line, e: eye, f: funnel, FL: funnel line, fp: funnel pocket, L1-5: lateral lines, ma: mantle, o: olfactory organ, va: ventral arm, VL: ventral line, vla: ventrolateral arm, VlL: ventrolateral line. (Lenz, 1995)

Biology Open • Supplementary information

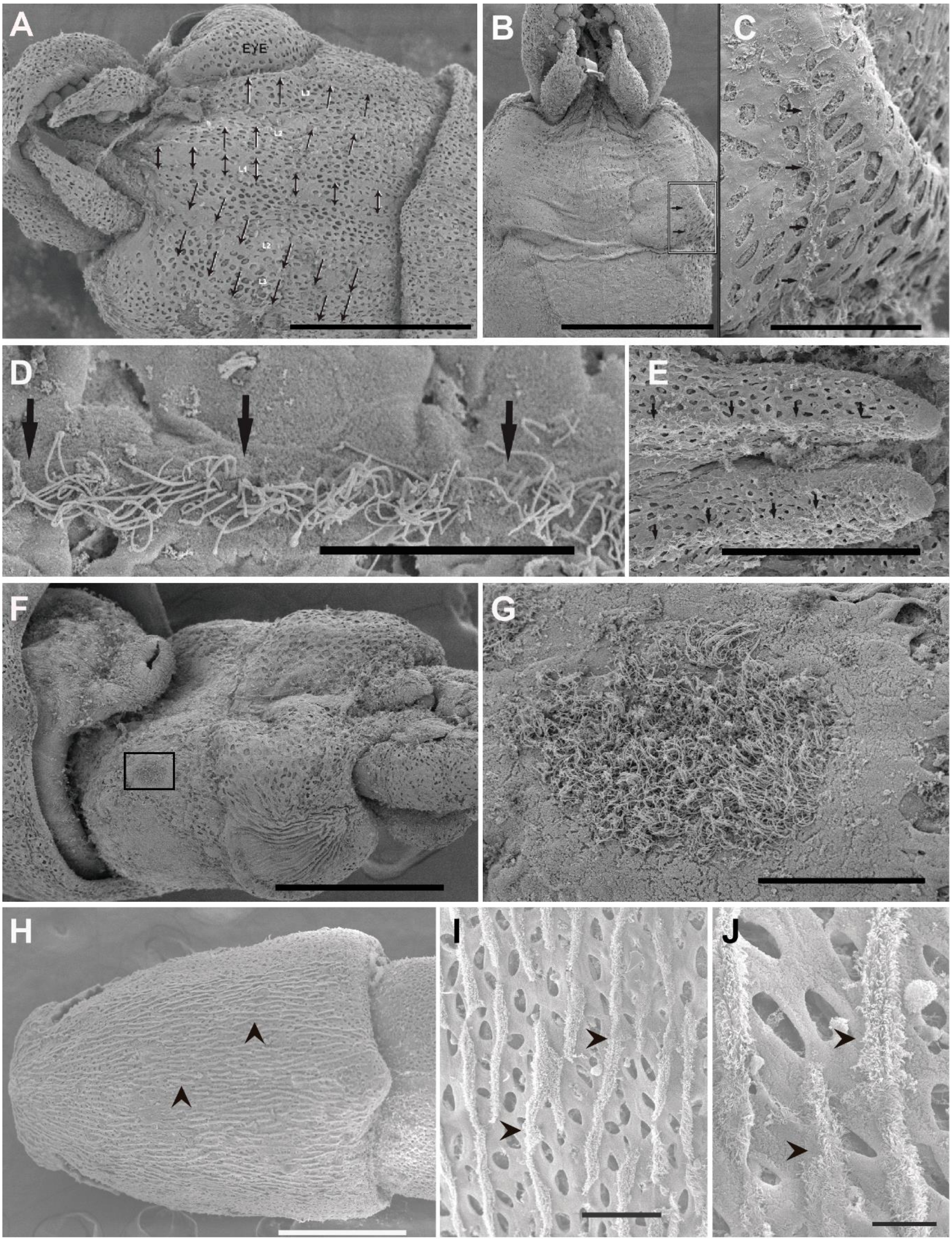

Fig. S2. SEM. Loligo vulgaris epidermal lines and accessory ciliated structures. Control animals. **A:** Dorsal side. Arrows show L1, L2 and L3. **B**: Dorsal view. Arrows indicate Line 3 running above the eye. **C**: Detail from B square. L3 (arrows). **D**: Arrows shows a detailed view from L1. Note the regular arrangements of the kinocilia hair cells. **E**: Ventral side. Arrows indicate the paired Line 5. **F:** Larva ventral view shows the olfactory organ (square) near the funnel. **G**: Detail from the square of F. Note the high density of the cilia of olfactory organ. **H**: Dorsal side. The squid mantle is covered by rows of ciliated cells (arrowheads). **I, J**: Details of H. Arrows point to cilia of the hair cell rows. **Scale bars: A, B, F, H** = 500 µm. **D, E** = 200 µm. **C** = 100 µm. **G, I** = 50 µm. **J** = 15 µm.

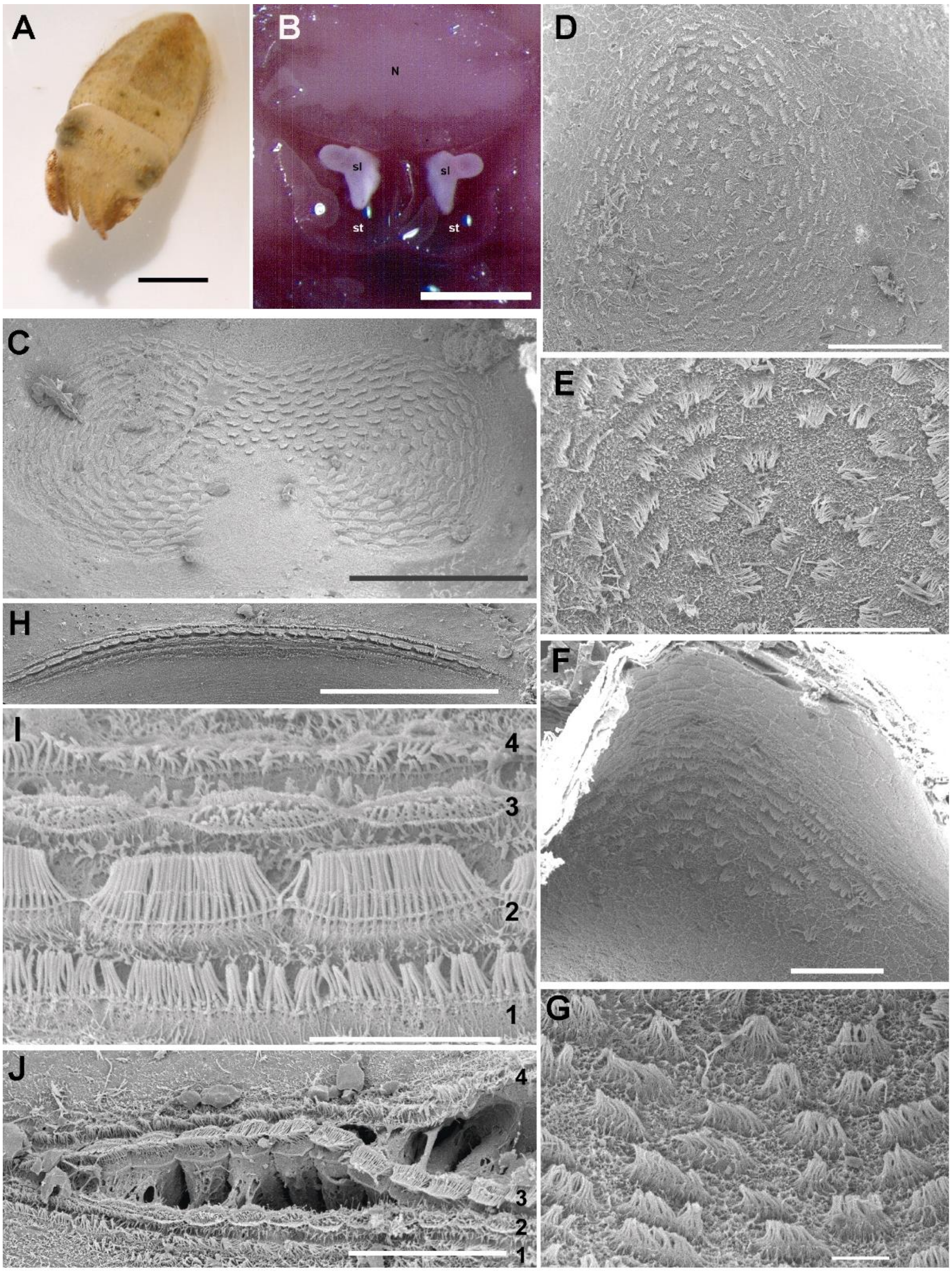

Fig. S3. A, B: LM. Photomicrograph of S. officinalis hatchling and its inner statocyst structure. C-J: SEM. S. officinalis hatchling inner statocyst morphology. Control hatchlings (A-I) and sacrificed immediately after sound exposure (J). A: Upper view of 1 day old cuttlefish. ***B:*** *Inner statocyst structure. Anterior view. Below the nervous sytem (N), the statocyst cavities have been opened transversally. Each statocyst cavity (st) shows the statolith (sl) attached to the macula statica princeps (msp).* ***C:*** *macula statica princeps*. The arrangements of the kinociliary groups of the hair cells are in regular lines following the epithelium shape. ***D:*** *Inferior macula neglecta (imn).* The hair cells are arranged in nearly concentric rings around a center. ***E:*** *Detail of D.* The ciliary groups of the *inm* are more sparsely distributed than those of the *msp*. ***F:*** *Superior macula neglecta (smn). T*he nearly half-circular shaped *smn* is visible. **G**: Detail of F. The ciliary groups of the *smn* are more sparsely distributed than those of the *msp.* **H**: Upper view of the crista system containing the four rows of sensory hair cells**. I:** Detail of H. The four rows of *crista* hair cells are visible. The hair cells from two of them are larger (1, 2) than those of the other two rows (3, 4). Surrounding the *crista*, other hair cells similar to the shorter ones of the main rows are found**. J**: On an exposed hatchling, the crista present obvious signs of damage, including bending kinocilia and cellular material extrusion. The epithelium is fractured between rows 2 and 3 and rows 3 and 4. Note that hair cells in row 3 are partially extruded**. Scale bars: A** = 0,5cm. **B, C, H** = 100 µm. **D** = 50 µm. **J** = 30 µm. **F** = 25 µm. **E** = 20 µm. **I**= 10 µm. **G**= 5 µm.

Biology Open • Supplementary information

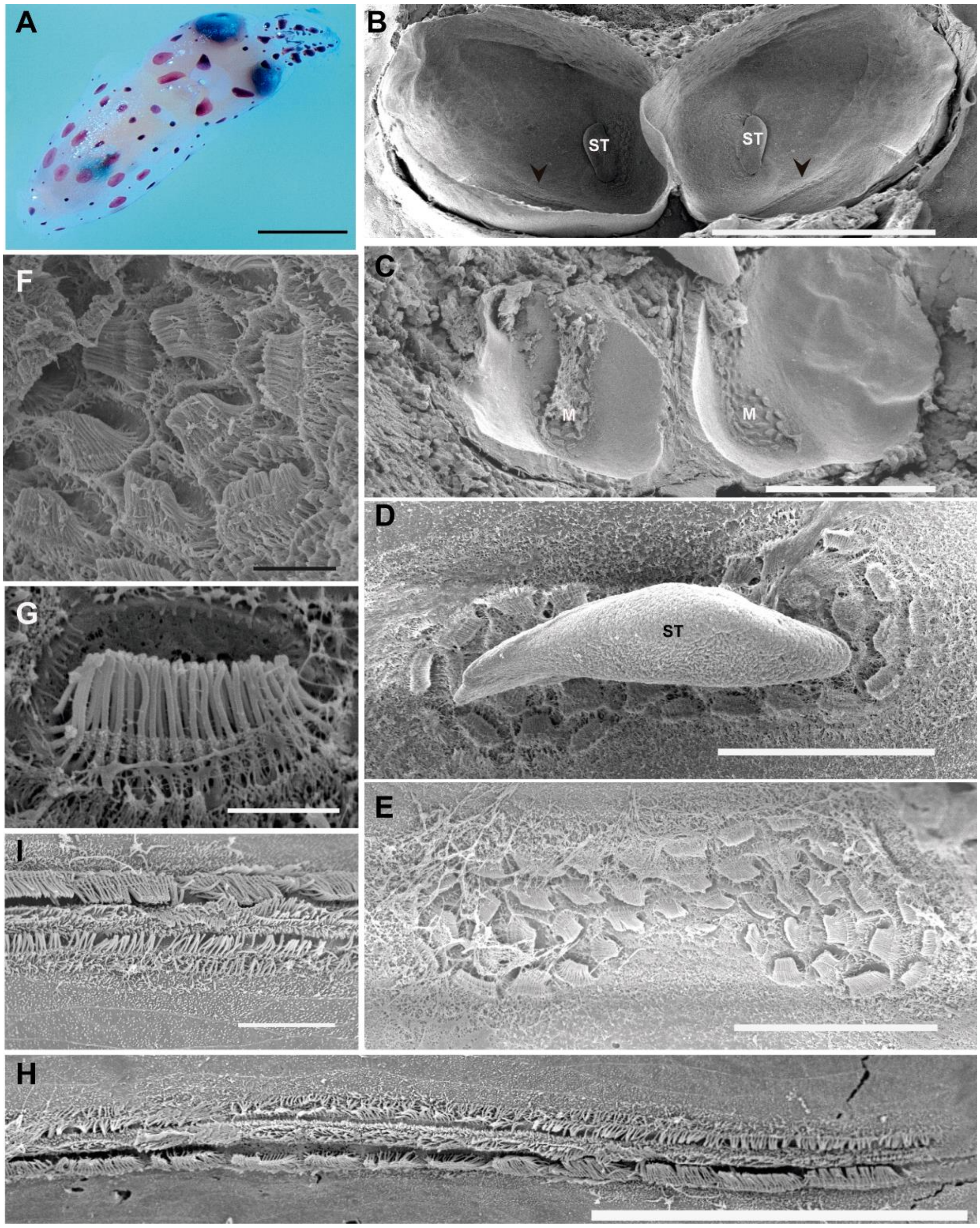

Fig. S4. A: LM. Photomicrograph of L vulgaris hatchling. B-I: SEM. L. vulgaris hatchling inner statocyst morphology. Control hatchlings. ***A****:* Upper view of hatchling common squids showing iridescent pigmentation due to the distribution of its epidermal cells. ***B****: Inner statocyst structure. Anterior view. The statocyst cavities have been opened transversally. Each statocyst cavity shows the statolith (ST) attached to the macula staticaprinceps (msp).* Arrowheads point to the only segment of crista visible. ***C****: The two msp (M) are visible on the open statocyst cavities. The statolith has been removed and some rests of the layer of mucus are visible on one of the macula (left in the image).* ***D****: The statolith (ST) attached to the macula is visible.* ***E****: In a macula where the statolith has been removed, t*he arrangements of the kinociliary groups of the hair cells are in regular lines following the epithelium shape are shown**.** ***F:*** Detail of the arrangements of the hair cells kinociliary groups in regular lines Note the organization of the *macula* epithelium is very similar in all decapodiforme species. **G*:*** Detail of a hair cell. Kinocilia and microvilli form elongated groups. Each kinociliary group represents a single hair cell. **H**: Upper view of the crista system containing the four rows of sensory hair cells. **I**: Detail of H. **Scale bars: A** = 1 mm. **B** = 200 µm. **C** = 100 µm. **H** = 50 µm. **D** = 30 µm. **E, I** = 10 µm. **F** = 5 µm. **G** = 3 µm.

	**OBSERVED EFFECTS**			
	**LATERAL LINE**		**STATOCYST (*macula statica princeps*)**	
TIME AFTER EXPOSURE	**0H**	**24H**	**0H**	**24H**
Sepia officinalis	**Hair cells:**<br>- exhibited **rests of their roots**<br>- had dramatically **lost almost all kinocilia**<br>- remaining **kinocilia bent, flaccid or fused**, or show **blebs**	The **same lesions** described at 0H presented an **increase** of severity	**Hair cells**:<br>- had **totally lost** their **kinocilia**<br>- showed **bent, flaccid or fused kinocilia**<br>- had their **apical pole extruded**<br>- the **expulsion of the cellular material** left **holes** in their base	The **same lesions** described at 0H presented an **increase** of severity
Loligo vulgaris	**Hair cells:**<br>- exhibited **rests of their roots**<br>- had dramatically **lost almost all kinocilia**<br>- remaining **kinocilia bent, flaccid or fused**, or show **blebs**	The **same lesions** described at 0H presented a **decrease** of severity	**Hair cells**:<br>- had **totally lost** their **kinocilia**<br>- showed **bent, flaccid or fused kinocilia**<br>- had their **apical pole extruded**<br>- the **expulsion of the cellular material** left **holes** in their base	The **same lesions** described at 0H presented an **increase** of severity
Illex coindetii	**Hair cells:**<br>- exhibited **rests of their roots**<br>- had dramatically **lost almost all kinocilia**<br>- remaining **kinocilia bent, flaccid or fused**, or show **blebs**	The **same lesions** described at 0H presented a **decrease** of severity	**Hair cells**:<br>- had **totally lost** their **kinocilia**<br>- showed **bent, flaccid or fused kinocilia**<br>- had their **apical pole extruded**<br>- the **expulsion of the cellular material** left **holes** in their base	The **same lesions** described at 0H presented an **increase** of severity

Table S1. Summary of the observed effects on the different sensory systems and species

Biology Open • Supplementary information

Table S2. Tables below show the summary of the larvae counts

MACULA SEPIA CONTROL		Ext-mis Cells/Macula Surfice	Cell Total number/ Macula Surfice
**1**	**Stato 1**	2,51168E-05	0,009745316
	**Stato 2**	0	0,014857143
**2**	**Stato 1**	0	0,014859406
	**Stato 2**	2,35905E-05	0,014861996
**3**	**Stato 1**	0	0,014756098
	**Stato 2**	4,34783E-05	0,014673913
**4**	**Stato 1**	0	0,014659091
	**Stato 2**	0	0,014765682
**5**	**Stato 1**	0	0,014862069
	**Stato 2**	0	0,01464
**6**	**Stato 1**	3,63372E-05	0,014135174
	**Stato 2**	0	0,014794521
**7**	**Stato 1**	0	0,014800885
	**Stato 2**	0	0,014876508
**8**	**Stato 1**	0	0,014817536
	**Stato 2**	0	0,01486592
**9**	**Stato 1**	2,40964E-05	0,014819277
	**Stato 2**	2,52366E-05	0,014132492
**10**	**Stato 1**	3,9268E-05	0,01474515
	**Stato 2**	0	0,014817383
**11**	**Stato 1**	0	0,01487069
	**Stato 2**	0	0,014799893
**12**	**Stato 1**	2,09732E-05	0,014869966
	**Stato 2**	0	0,014779559
**13**	**Stato 1**	0	0,014615385
	**Stato 2**	0	0,014647317
**14**	**Stato 1**	0	0,014573511
	**Stato 2**	0	0,01494898
**15**	**Stato 1**	0	0,015032859
	**Stato 2**	0	0,014855328
**16**	**Stato 1**	0	0,014869281
	**Stato 2**	0	0,014853826
**17**	**Stato 1**	0	0,01495222
	**Stato 2**	0	0,01483921
**18**	**Stato 1**	0	0,014940022
	**Stato 2**	0	0,014866784
**19**	**Stato 1**	2,0055E-05	0,014900828
	**Stato 2**	0	0,014860681
**20**	**Stato 1**	0	0,014883367
	**Stato 2**	0	0,014906518

Biology Open • Supplementary information

MACULA SEPIA 0h		Ext-mis Cells/Macula Surfice	Cell Total number/ Macula Surfice
1	Stato 1	0,004482759	0,013103448
	Stato 2	0,004148472	0,013318777
2	Stato 1	0,008096591	0,019507576
	Stato 2	0,004145078	0,013316062
3	Stato 1	0,005037069	0,013105102
	Stato 2	0,004470116	0,01310899
4	Stato 1	0,004467593	0,013101852
	Stato 2	0,00544	0,013088
5	Stato 1	0,004082083	0,013106796
	Stato 2	0,005062344	0,013167082
6	Stato 1	0,005053341	0,01316676
	Stato 2	0,004097268	0,013157895
7	Stato 1	0,004065888	0,013052544
	Stato 2	0,005433647	0,013061651
8	Stato 1	0,004491374	0,013087448
	Stato 2	0,004080146	0,013151184
9	Stato 1	0,004128702	0,013211845
	Stato 2	0,004118377	0,013141556
10	Stato 1	0,005026288	0,013038906
	Stato 2	0,004476861	0,013003018
11	Stato 1	0,004135241	0,013107932
	Stato 2	0,004153196	0,013106192
12	Stato 1	0,004166667	0,013106061
	Stato 2	0,004077922	0,013090909
13	Stato 1	0,004130481	0,013111629
	Stato 2	0,004484605	0,013052209
14	Stato 1	0,004154229	0,013134328
	Stato 2	0,005486542	0,013167702
15	Stato 1	0,004132231	0,013105077
	Stato 2	0,004116022	0,013038674
16	Stato 1	0,005203785	0,013282387
	Stato 2	0,004144687	0,013112283
17	Stato 1	0,003980655	0,013095238
	Stato 2	0,004124534	0,01309207
18	Stato 1	0,004149473	0,013136176
	Stato 2	0,005036995	0,013118953
19	Stato 1	0,004113046	0,01317184
	Stato 2	0,005547189	0,013353414
20	Stato 1	0,004089219	0,013110285
	Stato 2	0,00406885	0,013094299

Biology Open • Supplementary information

MACULA SEPIA 24h		Ext-mis Cells/Macula Surfice	Cell Total number/ Macula Surfice
1	Stato 1	0,005264322	0,012231807
	Stato 2	0,003785967	0,012216052
2	Stato 1	0,005801606	0,012224812
	Stato 2	0,007175141	0,012231638
3	Stato 1	0,004058175	0,01222144
	Stato 2	0,00660461	0,012167553
4	Stato 1	0,006479156	0,011024611
	Stato 2	0,005805593	0,012223702
5	Stato 1	0,005282555	0,012223587
	Stato 2	0,003845363	0,012235246
6	Stato 1	0,004092015	0,012231807
	Stato 2	0,00411985	0,0122155
7	Stato 1	0,007075472	0,012224843
	Stato 2	0,005801861	0,012233169
8	Stato 1	0,006246222	0,012270804
	Stato 2	0,006665233	0,01227693
9	Stato 1	0,007201859	0,012235416
	Stato 2	0,005863539	0,012233475
10	Stato 1	0,005251142	0,01217656
	Stato 2	0,003800883	0,012211869
11	Stato 1	0,006669901	0,012151346
	Stato 2	0,004138889	0,01225
12	Stato 1	0,006737731	0,012288817
	Stato 2	0,006699147	0,012228989
13	Stato 1	0,005332693	0,01222676
	Stato 2	0,006683971	0,012221501
14	Stato 1	0,005300926	0,012291667
	Stato 2	0,006784915	0,012261064
15	Stato 1	0,006634615	0,012211538
	Stato 2	0,006673961	0,01221736
16	Stato 1	0,006651463	0,012200684
	Stato 2	0,006708005	0,012232244
17	Stato 1	0,006746295	0,012298164
	Stato 2	0,006752232	0,012248884
18	Stato 1	0,006692112	0,012290076
	Stato 2	0,006823028	0,012233475
19	Stato 1	0,006830986	0,012276995
	Stato 2	0,006768398	0,012207728
20	Stato 1	0,007220736	0,01224028
	Stato 2	0,007273501	0,012292641

LAT LIN SEPIA CONTROL		Ext-mis Cells/Macula Surfice	Cell Total number/ Lat lin Surfice
1	L1a	0	0,001724138
	L1b	2,87356E-05	0,001810345
2	L1a	2,93686E-05	0,001820852
	L1b	0	0,001791483
3	L1a	0	0,001785714
	L1b	0	0,001815476
4	L1a	3,10078E-05	0,001705426
	L1b	0	0,001736434
5	L1a	0	0,001698113
	L1b	3,14465E-05	0,00172956
6	L1a	5,35475E-05	0,001874163
	L1b	2,67738E-05	0,001740295
7	L1a	0	0,001690141
	L1b	0	0,001690141
8	L1a	0	0,001865569
	L1b	2,74348E-05	0,001893004
9	L1a	0	0,001704036
	L1b	0	0,001674141
10	L1a	4,90196E-05	0,001715686
	L1b	0	0,001740196
11	L1a	2,26757E-05	0,001791383
	L1b	4,53515E-05	0,001882086
12	L1a	0	0,001735849
	L1b	0	0,001735849
13	L1a	0	0,001801802
	L1b	6,75676E-05	0,001824324
14	L1a	0	0,001789322
	L1b	0	0,001731602
15	L1a	0	0,001740413
	L1b	2,94985E-05	0,001769912
16	L1a	0	0,001748148
	L1b	0	0,001777778
17	L1a	0	0,001682243
	L1b	0	0,001713396
18	L1a	2,92398E-05	0,001783626
	L1b	0	0,001783626
19	L1a	0	0,001704718
	L1b	0	0,001704718
20	L1a	0	0,001704036
	L1b	2,98954E-05	0,001704036

LAT LIN SEPIA 0h		Ext-mis Cells/Macula Surfice	Cell Total number/ Lat lin Surfice
1	L1a	0,000225564	0,001804511
	L1b	0,000250627	0,001879699
2	L1a	0,000408163	0,001791383
	L1b	0,000453515	0,001927438
3	L1a	0,000434109	0,001829457
	L1b	0,000403101	0,001891473
4	L1a	0,000267857	0,001875
	L1b	0,000267857	0,001934524
5	L1a	0,000452489	0,001809955
	L1b	0,000392157	0,001840121
6	L1a	0,000438957	0,001783265
	L1b	0,000438957	0,001838134
7	L1a	0,000442338	0,001832543
	L1b	0,000410742	0,00192733
8	L1a	0,000329531	0,001774398
	L1b	0,000430925	0,001825095
9	L1a	0,00043379	0,001780822
	L1b	0,000456621	0,001803653
10	L1a	0,00045977	0,001864623
	L1b	0,000383142	0,001787995
11	L1a	0,00042735	0,001766382
	L1b	0,00034188	0,001794872
12	L1a	0,000524017	0,001950509
	L1b	0,000494905	0,001892285
13	L1a	0,00036036	0,001801802
	L1b	0,00045045	0,001831832
14	L1a	0,000344288	0,001846635
	L1b	0,000469484	0,001940532
15	L1a	0,000473934	0,001864139
	L1b	0,000442338	0,001895735
16	L1a	0,000483871	0,001801075
	L1b	0,000456989	0,001801075
17	L1a	0,000377358	0,001855346
	L1b	0,000283019	0,001918239
18	L1a	0,000510753	0,001801075
	L1b	0,000430108	0,001827957
19	L1a	0,000440529	0,001938326
	L1b	0,000499266	0,001938326
20	L1a	0,000369004	0,001820418
	L1b	0,000442804	0,001795818

LAT LIN SEPIA 24h		Ext-mis Cells/Macula Surfice	Cell Total number/ Lat lin Surfice
1	L1a	0,000859259	0,001837037
	L1b	0,00077037	0,001866667
2	L1a	0,000757576	0,001848485
	L1b	0,000666667	0,001818182
3	L1a	0,00079646	0,001887906
	L1b	0,000737463	0,001828909
4	L1a	0,000746888	0,001881051
	L1b	0,000857538	0,001991701
5	L1a	0,000597484	0,001823899
	L1b	0,000786164	0,001855346
6	L1a	0,000805031	0,001861635
	L1b	0,000754717	0,001886792
7	L1a	0,000796359	0,001865757
	L1b	0,000841866	0,001934016
8	L1a	0,000811153	0,001825095
	L1b	0,000811153	0,001875792
9	L1a	0,000821918	0,001894977
	L1b	0,000753425	0,001917808
10	L1a	0,000804598	0,001896552
	L1b	0,000833333	0,001867816
11	L1a	0,000792952	0,00185022
	L1b	0,000734214	0,00185022
12	L1a	0,000737463	0,001828909
	L1b	0,000884956	0,001917404
13	L1a	0,000825688	0,001834862
	L1b	0,000672783	0,001834862
14	L1a	0,000815138	0,001834061
	L1b	0,000727802	0,001863173
15	L1a	0,000761035	0,001856925
	L1b	0,000852359	0,001826484
16	L1a	0,000885201	0,001908714
	L1b	0,001051176	0,001908714
17	L1a	0,000761035	0,001796043
	L1b	0,000669711	0,001826484
18	L1a	0,000766284	0,001813538
	L1b	0,000740741	0,00183908
19	L1a	0,000824742	0,00185567
	L1b	0,000756014	0,001901489
20	L1a	0,000880503	0,001861635
	L1b	0,000830189	0,001861635

Biology Open • Supplementary information

MACULA LOLIGO CONTROL		Ext-mis Cells/Macula Surfice	Cell Total number/ Macula Surfice
**1**	**Stato 1**	0	0,023595506
	**Stato 2**	0	0,022857143
**2**	**Stato 1**	0	0,023958333
	**Stato 2**	0	0,023152709
**3**	**Stato 1**	0,00027027	0,016756757
	**Stato 2**	0	0,0168
**4**	**Stato 1**	0	0,023245614
	**Stato 2**	0	0,020642202
**5**	**Stato 1**	0	0,015760041
	**Stato 2**	0	0,016455696
**6**	**Stato 1**	0	0,015737874
	**Stato 2**	0	0,022893327
**7**	**Stato 1**	0	0,023529412
	**Stato 2**	0	0,023626374
**8**	**Stato 1**	0	0,023650386
	**Stato 2**	0,000273973	0,016712329
**9**	**Stato 1**	0,000440529	0,023348018
	**Stato 2**	0	0,024185068
**10**	**Stato 1**	0	0,023209877
	**Stato 2**	0,000257732	0,015721649
**11**	**Stato 1**	0	0,015836526
	**Stato 2**	0	0,024109589
**12**	**Stato 1**	0,000398406	0,022310757
	**Stato 2**	0	0,02393617
**13**	**Stato 1**	0	0,023834197
	**Stato 2**	0	0,023215322
**14**	**Stato 1**	0	0,022169811
	**Stato 2**	0,000393236	0,022414471
**15**	**Stato 1**	0	0,023517382
	**Stato 2**	0	0,022917831
**16**	**Stato 1**	0	0,02310231
	**Stato 2**	0	0,023650386
**17**	**Stato 1**	0	0,024309392
	**Stato 2**	0	0,022688599
**18**	**Stato 1**	0,00044287	0,023914969
	**Stato 2**	0	0,022803115
**19**	**Stato 1**	0	0,024
	**Stato 2**	0	0,024109589
**20**	**Stato 1**	0	0,022611645
	**Stato 2**	0	0,02301496

Biology Open • Supplementary information

MACULA LOLIGO 0h		Ext-mis Cells/Macula Surfice	Cell Total number/ Macula Surfice
1	Stato 1	0,002948403	0,023587224
	Stato 2	0,001827676	0,015665796
2	Stato 1	0,001785714	0,015816327
	Stato 2	0,002708559	0,023293608
3	Stato 1	0,002745098	0,021960784
	Stato 2	0,002617801	0,02408377
4	Stato 1	0,002597403	0,023896104
	Stato 2	0,001166181	0,023906706
5	Stato 1	0,002962963	0,023209877
	Stato 2	0,002371542	0,022134387
6	Stato 1	0,002527806	0,023255814
	Stato 2	0,000574713	0,022988506
7	Stato 1	0,002583979	0,023255814
	Stato 2	0,002072539	0,023834197
8	Stato 1	0,001624256	0,023822415
	Stato 2	0,001142857	0,022857143
9	Stato 1	0,003160271	0,023476298
	Stato 2	0,001675978	0,023463687
10	Stato 1	0,002104156	0,024197791
	Stato 2	0,001089325	0,023965142
11	Stato 1	0,001129944	0,023163842
	Stato 2	0,001146132	0,022922636
12	Stato 1	0,001120448	0,023529412
	Stato 2	0,002383222	0,022878932
13	Stato 1	0,002512563	0,02361809
	Stato 2	0,001965602	0,023095823
14	Stato 1	0,001623377	0,016774892
	Stato 2	0,001866667	0,0168
15	Stato 1	0,002597403	0,023376623
	Stato 2	0,002072539	0,023834197
16	Stato 1	0,001797753	0,023370787
	Stato 2	0,001129944	0,023728814
17	Stato 1	0,001728111	0,023041475
	Stato 2	0,00195122	0,022926829
18	Stato 1	0,002298851	0,022988506
	Stato 2	0,002191781	0,023561644
19	Stato 1	0,002558854	0,023541453
	Stato 2	0,001617251	0,01671159
20	Stato 1	0,002216312	0,023492908
	Stato 2	0,002296211	0,023536165

Biology Open • Supplementary information

MACULA LOLIGO 24h		Ext-mis Cells/Macula Surfice	Cell Total number/ Macula Surfice
1	Stato 1	0,005837712	0,023934618
	Stato 2	0,005393258	0,021573034
2	Stato 1	0,005759162	0,023560209
	Stato 2	0,00450676	0,023034552
3	Stato 1	0,003916449	0,016449086
	Stato 2	0,00398293	0,017638691
4	Stato 1	0,004357298	0,023965142
	Stato 2	0,005301205	0,022168675
5	Stato 1	0,003664921	0,015968586
	Stato 2	0,004078511	0,016314045
6	Stato 1	0,003145478	0,015727392
	Stato 2	0,005502751	0,023011506
7	Stato 1	0,005287897	0,023501763
	Stato 2	0,005420054	0,023306233
8	Stato 1	0,005112474	0,023517382
	Stato 2	0,003588187	0,016560861
9	Stato 1	0,005922551	0,023690205
	Stato 2	0,005007511	0,023034552
10	Stato 1	0,004943154	0,023232823
	Stato 2	0,003580563	0,015601023
11	Stato 1	0,003831418	0,015836526
	Stato 2	0,005524862	0,024309392
12	Stato 1	0,004011231	0,022061773
	Stato 2	0,006319115	0,024223275
13	Stato 1	0,006799163	0,023535565
	Stato 2	0,005214368	0,023174971
14	Stato 1	0,004662005	0,022377622
	Stato 2	0,00461361	0,021914648
15	Stato 1	0,005583756	0,023857868
	Stato 2	0,005022321	0,022879464
16	Stato 1	0,005242464	0,023591088
	Stato 2	0,005197505	0,023388773
17	Stato 1	0,005387931	0,02424569
	Stato 2	0,005157593	0,022922636
18	Stato 1	0,005719314	0,024197096
	Stato 2	0,005561735	0,023359288
19	Stato 1	0,005260389	0,024197791
	Stato 2	0,00607064	0,024282561
20	Stato 1	0,005649718	0,02259887
	Stato 2	0,005172414	0,022988506

Biology Open • Supplementary information

LAT LIN LOLIGO CONTROL		Ext-mis Cells/Macula Surfice	Cell Total number/ Lat lin Surfice
1	L1a	0,000140647	0,004781997
	L1b	0	0,00464135
2	L1a	0,000152672	0,005496183
	L1b	0	0,005343511
3	L1a	0,000136986	0,004520548
	L1b	0,000136986	0,004520548
4	L1a	0	0,004428571
	L1b	0	0,004428571
5	L1a	0,000143062	0,004005722
	L1b	0,000143062	0,004148784
6	L1a	0	0,004275862
	L1b	0	0,004551724
7	L1a	0	0,003900709
	L1b	0	0,004078014
8	L1a	0	0,004557292
	L1b	0	0,0046875
9	L1a	0	0,004230317
	L1b	0	0,004347826
10	L1a	0,000144092	0,004034582
	L1b	0	0,004034582
11	L1a	0,00014245	0,004700855
	L1b	0	0,004700855
12	L1a	0	0,00530303
	L1b	0	0,005151515
13	L1a	0,000138889	0,004444444
	L1b	0	0,004305556
14	L1a	0,000143062	0,004434907
	L1b	0	0,004434907
15	L1a	0	0,004125178
	L1b	0	0,00455192
16	L1a	0	0,004109589
	L1b	0	0,004520548
17	L1a	0	0,003928571
	L1b	0	0,004107143
18	L1a	0	0,004545455
	L1b	0	0,004675325
19	L1a	0	0,004302326
	L1b	0,000116279	0,004418605
20	L1a	0,000142653	0,004279601
	L1b	0	0,004136947

Biology Open • Supplementary information

LAT LIN LOLIGO 0h		Ext-mis Cells/Macula Surfice	Cell Total number/ Lat lin Surfice
1	L1a	0,002659574	0,004388298
	L1b	0,002526596	0,004521277
2	L1a	0,000967352	0,004474002
	L1b	0,001088271	0,004353083
3	L1a	0,001507538	0,004145729
	L1b	0,001130653	0,004145729
4	L1a	0,002112676	0,004366197
	L1b	0,002112676	0,004366197
5	L1a	0,003067485	0,005521472
	L1b	0,00291411	0,005368098
6	L1a	0,001481481	0,004197531
	L1b	0,000987654	0,004567901
7	L1a	0,001570681	0,004581152
	L1b	0,00117801	0,004712042
8	L1a	0,002026049	0,004341534
	L1b	0,002315485	0,004341534
9	L1a	0,001812689	0,005135952
	L1b	0,002870091	0,005287009
10	L1a	0,002028986	0,004347826
	L1b	0,002173913	0,004202899
11	L1a	0,001546392	0,004123711
	L1b	0,001718213	0,004295533
12	L1a	0,0024	0,0044
	L1b	0,002533333	0,0044
13	L1a	0,001219512	0,004390244
	L1b	0,001097561	0,004390244
14	L1a	0,001511335	0,004156171
	L1b	0,001259446	0,004156171
15	L1a	0,001974612	0,004513399
	L1b	0,002115656	0,004654443
16	L1a	0,00229709	0,004594181
	L1b	0,002143951	0,004594181
17	L1a	0,001376721	0,004505632
	L1b	0,001251564	0,004630788
18	L1a	0,00155642	0,004539559
	L1b	0,001686122	0,004669261
19	L1a	0,002460203	0,00463097
	L1b	0,00260492	0,004486252
20	L1a	0,002114804	0,004531722
	L1b	0,002265861	0,004531722

Biology Open • Supplementary information

LAT LIN LOLIGO 24h		Ext-mis Cells/Macula Surfice	Cell Total number/ Lat lin Surfice
**1**	**L1a**	0,000442478	0,004572271
	**L1b**	0,000442478	0,004277286
**2**	**L1a**	0,001042874	0,004519119
	**L1b**	0,000811124	0,00405562
**3**	**L1a**	0,00046729	0,004672897
	**L1b**	0,00046729	0,004361371
**4**	**L1a**	0,000453858	0,004387292
	**L1b**	0,000453858	0,004387292
**5**	**L1a**	0,001843972	0,004113475
	**L1b**	0,001134752	0,004255319
**6**	**L1a**	0,001212121	0,004121212
	**L1b**	0,001212121	0,004484848
**7**	**L1a**	0,000659631	0,00474934
	**L1b**	0,000923483	0,00474934
**8**	**L1a**	0,001097394	0,004115226
	**L1b**	0,001097394	0,003978052
**9**	**L1a**	0,000410397	0,004103967
	**L1b**	0,000410397	0,004103967
**10**	**L1a**	0,001762115	0,003817915
	**L1b**	0,001174743	0,004111601
**11**	**L1a**	0,001176471	0,003676471
	**L1b**	0,001323529	0,004117647
**12**	**L1a**	0,000582242	0,004366812
	**L1b**	0,000873362	0,004366812
**13**	**L1a**	0,000441176	0,004411765
	**L1b**	0,000441176	0,004411765
**14**	**L1a**	0,001040462	0,004393064
	**L1b**	0,000809249	0,00416185
**15**	**L1a**	0,00046875	0,0046875
	**L1b**	0,00046875	0,004375
**16**	**L1a**	0,000454545	0,00469697
	**L1b**	0,000454545	0,004545455
**17**	**L1a**	0,001428571	0,004142857
	**L1b**	0,001142857	0,004285714
**18**	**L1a**	0,001226994	0,004294479
	**L1b**	0,001226994	0,004417178
**19**	**L1a**	0,000657895	0,004736842
	**L1b**	0,000921053	0,004736842
**20**	**L1a**	0,00109589	0,004109589
	**L1b**	0,00109589	0,003972603

Biology Open • Supplementary information

MACULA ILLEX CONTROL		Ext-mis Cells/Macula Surfice	Cell Total number/ Macula Surfice
1	Stato 1	0	0,02464332
	Stato 2	0	0,024719101
2	Stato 1	0,001149425	0,022988506
	Stato 2	0	0,024242424
3	Stato 1	0	0,024137931
	Stato 2	0,001123596	0,024719101
4	Stato 1	0	0,02962963
	Stato 2	0	0,030909091
5	Stato 1	0	0,025301205
	Stato 2	0	0,024324324
6	Stato 1	0	0,025862069
	Stato 2	0	0,024657534
7	Stato 1	0	0,023809524
	Stato 2	0	0,02425107
8	Stato 1	0	0,025333333
	Stato 2	0	0,024836601
9	Stato 1	0	0,024516129
	Stato 2	0	0,023448276
10	Stato 1	0	0,025062657
	Stato 2	0	0,024161074

MACULA ILLEX 0h		Ext-mis Cells/Macula Surfice	Cell Total number/ Macula Surfice
1	Stato 1	0,003076923	0,024615385
	Stato 2	0,002702703	0,024324324
2	Stato 1	0,002849003	0,024216524
	Stato 2	0,003030303	0,024242424
3	Stato 1	0,002659574	0,02393617
	Stato 2	0,003159558	0,023696682
4	Stato 1	0	0,029090909
	Stato 2	0,003174603	0,026984127
5	Stato 1	0,002590674	0,023316062
	Stato 2	0,003205128	0,025641026
6	Stato 1	0,003225806	0,024193548
	Stato 2	0,0016	0,0256
7	Stato 1	0,002781641	0,02364395
	Stato 2	0,002853067	0,02425107
8	Stato 1	0,002666667	0,026666667
	Stato 2	0,002932551	0,024926686
9	Stato 1	0,002580645	0,024516129
	Stato 2	0,001503759	0,022556391
10	Stato 1	0,001492537	0,023880597
	Stato 2	0,003100775	0,023255814

Biology Open • Supplementary information

MACULA ILLEX 24h		Ext-mis Cells/Macula Surfice	Cell Total number/ Macula Surfice
1	Stato 1	0,005369128	0,025503356
	Stato 2	0,004615385	0,024615385
2	Stato 1	0,005970149	0,023880597
	Stato 2	0,004379562	0,024817518
3	Stato 1	0,005369128	0,024161074
	Stato 2	0,004477612	0,023880597
4	Stato 1	0,004761905	0,025396825
	Stato 2	0,005797101	0,024637681
5	Stato 1	0,005563282	0,025034771
	Stato 2	0,004918033	0,024590164
6	Stato 1	0,006451613	0,024193548
	Stato 2	0,004477612	0,023880597
7	Stato 1	0,005517241	0,024827586
	Stato 2	0,004761905	0,025396825
8	Stato 1	0,005333333	0,024
	Stato 2	0,004545455	0,024242424
9	Stato 1	0,006451613	0,024516129
	Stato 2	0,006269592	0,023510972
10	Stato 1	0,00433526	0,023121387
	Stato 2	0,004411765	0,022058824

LAT LIN ILLEX CONTROL		Ext-mis Cells/Macula Surfice	Cell Total number/ Lat lin Surfice
1	L1a	7,75194E-05	0,008449612
	L1b	0	0,007596899
2	L1a	9,87654E-05	0,008641975
	L1b	4,93827E-05	0,008444444
3	L1a	0	0,008461538
	L1b	0	0,007615385
4	L1a	0	0,008578431
	L1b	9,80392E-05	0,00872549
5	L1a	5,98802E-05	0,008862275
	L1b	5,98802E-05	0,008383234
6	L1a	5,08906E-05	0,008651399
	L1b	0	0,008549618
7	L1a	0	0,008623377
	L1b	0	0,008571429
8	L1a	0	0,008145455
	L1b	0	0,008581818
9	L1a	0	0,008307692
	L1b	0	0,007692308
10	L1a	4,81928E-05	0,008578313
	L1b	0,000144578	0,008771084

Biology Open • Supplementary information

LAT LIN ILLEX 0h		Ext-mis Cells/Macula Surfice	Cell Total number/ Lat lin Surfice
1	L1a	0,001757106	0,00878553
	L1b	0,001705426	0,008527132
2	L1a	0,001557632	0,008411215
	L1b	0,001744548	0,008785047
3	L1a	0,001958763	0,008814433
	L1b	0,001907216	0,008556701
4	L1a	0,00212987	0,008779221
	L1b	0,002025974	0,008675325
5	L1a	0,001769231	0,008461538
	L1b	0,001846154	0,007692308
6	L1a	0,001707317	0,008536585
	L1b	0,001853659	0,008682927
7	L1a	0,001746988	0,008795181
	L1b	0,001927711	0,008373494
8	L1a	0,001794872	0,008666667
	L1b	0,001948718	0,008717949
9	L1a	0,001789474	0,008684211
	L1b	0,001894737	0,008578947
10	L1a	0,001925926	0,008296296
	L1b	0,001851852	0,007555556

LAT LIN ILLEX 24h		Ext-mis Cells/Macula Surfice	Cell Total number/ Lat lin Surfice
1	L1a	0,000784314	0,007764706
	L1b	0,000941176	0,007843137
2	L1a	0,000764588	0,007806841
	L1b	0,000724346	0,007927565
3	L1a	0,000892857	0,00875
	L1b	0,000952381	0,008333333
4	L1a	0,000757576	0,008686869
	L1b	0,000909091	0,008737374
5	L1a	0,000883117	0,008519481
	L1b	0,000883117	0,008779221
6	L1a	0,000872727	0,007563636
	L1b	0,001163636	0,008218182
7	L1a	0,000772201	0,008416988
	L1b	0,000772201	0,007722008
8	L1a	0,000835381	0,008599509
	L1b	0,000933661	0,008501229
9	L1a	0,000926641	0,008494208
	L1b	0,000849421	0,008108108
10	L1a	0,000906921	0,00849642
	L1b	0,000954654	0,008591885

Biology Open • Supplementary information
